# Discovery of benzyl carbamate inhibitors of coronavirus M^pro^ enzymes from a legacy collection of cysteine protease inhibitors

**DOI:** 10.1080/14756366.2025.2585619

**Published:** 2025-11-17

**Authors:** Mateus Sá Magalhães Serafim, Thales Kronenberger, Karol R. Francisco, Erik Vinicius de Sousa Reis, Ellen Gonçalves de Oliveira, Fernanda Kelly Marcelino e Oliveira, Isadora Serraglio Fortes, Thaís Helena Maciel Fernandes, Elany Barbosa da Silva, Pavla Fajtova, Danielle E. Skinner, Rafay O. Syed, Jair Lage de Siqueira-Neto, Antti Poso, Bruno Eduardo Fernandes Mota, Jordana Grazziela Alves Coelho-dos-Reis, Jônatas Santos Abrahão, Vinícius Gonçalves Maltarollo, Anthony J. O’Donoghue, Conor R. Caffrey

**Affiliations:** ^a^ Center for Discovery and Innovation in Parasitic Diseases, Skaggs School of Pharmacy and Pharmaceutical Sciences, University of California San Diego, La Jolla, CA, USA; ^b^ Department of Microbiology, Institute of Biological Sciences, Federal University of Minas Gerais, Minas Gerais, Brazil; ^c^ Departament of Clinical and Toxicological Analysis, Faculty of Pharmacy, Federal University of Minas Gerais, Minas Gerais, Brazil; ^d^ Institut für Medizinische Mikrobiologie und Hygiene and DZIF (Center for Infection Research) Partner-site Tübingen, Tübingen, Germany; ^e^ School of Pharmacy, Faculty of Health Sciences, University of Eastern Finland, Kuopio, Finland; ^f^ Faculty of Pharmaceutical Sciences, Federal University of Rio Grande do Sul, Porto Alegre, Rio Grande do Sul, Brazil; ^g^ Pharmaceutical Sciences Graduate Program, Federal University of Rio Grande do Sul, Porto Alegre, Rio Grande do Sul, Brazil; ^h^ Department of Raw Material, Faculty of Pharmacy, Federal University of Rio Grande do Sul, Porto Alegre, Rio Grande do Sul, Brazil; ^i^ Institute of Pharmacy, Pharmaceutical/Medicinal Chemistry and Tübingen Center for Academic Drug Discovery (TüCAD2), Eberhard Karls University Tübingen, Tübingen, Germany; ^j^ Department of Pharmaceutical Products, Faculty of Pharmacy, Federal University of Minas Gerais, Minas Gerais, Brazil

**Keywords:** COVID-19, cysteine protease, human cathepsin L, M^pro^, SARS-CoV-2

## Abstract

The constant emergence of SARS-CoV-2 resistance drives the search for new antivirals. We screened the SARS-CoV-2 cysteine proteases, the main protease (M^pro^) and papain-like protease (PL^pro^), with 141 peptidyl and peptidomimetic inhibitors designed to target a trypanosome cysteine protease. Five compounds (**1a**–**5a**) inhibited M^pro^ (IC_50_ of 0.1601–16.42 µM), whereas none inhibited PL^pro^. Compounds **1a**–**4a** inhibited human cathepsin L (hCatL; 0.184–10.74 µM), which is important for viral entry into human cells. Compounds **1a** and **5a**, and its synthesised (R,S) enantiomer, **5b**, which share a benzyl carbamate moiety, inhibited the M^pro^ of SARS-CoV/MERS-CoV (0.0732–0.8295 µM). The three compounds were biochemically characterised as covalent reversible inhibitors. Compounds **5a** and **5b**, which contain vinyl ketone warheads, were specific for M^pro^, and this behaviour was supported by covalent and noncovalent computational simulations. This study highlights the importance of revisiting legacy assets to identify starting points for new antiviral drugs.

## Introduction

As of July 2025, approximately 778.4 million severe acute respiratory syndrome-related coronavirus 2 (SARS-CoV-2) infections have been reported worldwide, with over 7 million deaths due to coronavirus disease 2019 (COVID-19)^1^. The emergency approval of vaccines (e.g. mRNA^2^) and the repurposing of pharmacotherapies involving monoclonal antibodies and immunomodulatory drugs^3^, as well as the development of specific anti-coronavirus drugs^4^, have been pivotal to tackling the COVID-19 pandemic^5^. Regarding drug repurposing strategies, remdesivir, an adenosine analogue drug that was originally developed to treat hepatitis C virus^6^ and syncytial respiratory virus^7^ infections^8^, was successfully used to treat Ebola^9^^,^^10^ before being repurposed for SARS-CoV-2 infections^11^^,^^12^. Remdesivir has broad antiviral activity against both these RNA viruses and other highly pathogenic coronaviruses, such as SARS-CoV and MERS-CoV^13–15^.

In addition to repurposing strategies^16^^,^^17^, the design and discovery of new antivirals^18^^,^^19^ have also helped reduce COVID-19-related deaths^20^^,^^21^. Three oral antiviral drugs are now approved to treat COVID-19; the RNA polymerase inhibitor, molnupiravir^22^, and the two reversible inhibitors of the SARS-CoV-2 main protease (M^pro^), ensitrelvir^23^ and ritonavir-boosted nirmatrelvir^24^, the latter of which is marketed under the brand name, Paxlovid^TM^. However, nirmatrelvir has limitations, being recommended only for patients with mild-to-moderate COVID-19 symptoms^25^ while also exhibiting detrimental drug-drug interactions due to inhibition of CYP3A4^26–28^. Further, M^pro^ mutations associated with drug resistance^29^ to both ensitrelvir^30^ and nirmatrelvir^31^^,^^32^ have been reported, in addition to those which occur naturally for nirmatrelvir^33^, raising concerns regarding the drugs’ continued utility to treat SARS-CoV-2 infection^32^^,^^34^, and emphasising the need for additional antiviral drug options^3^. The same is true for the nirmatrelvir derivative and clinical candidate, PF-07817883 (ibuzatrelvir), for which sensitivity to M^pro^ mutations has already been reported *in vitro*^35^.

M^pro^ and the papain-like protease (PL^pro^), are viral cysteine proteases essential for SARS-CoV-2 replication^36^. In addition, human cathepsin L (hCatL) is important for viral entry^37^. All three enzymes are classified as cysteine proteases^38^^,^^39^ and small molecule inhibition of the reactive cysteine nucleophile in these enzymes is an established therapeutic strategy^39^^,^^40^, inhibiting virus polyprotein cleavage^41^^,^^42^ by M^pro^ or PL^pro^, or preventing entry of the virus into host cells in the case of hCatL^43^. Inhibition of cysteine proteases in eukaryotic parasites is also an established therapeutic strategy^44^, such as cruzain, a well-described drug target in *Trypanosoma cruzi*, which causes Chagas disease^45^^,^^46^. Between 1997 and 2012, a collection of small molecules designed to inhibit cruzain was synthesised as part of a drug development campaign to derive potency and specificity^47–51^. Considering, therefore, that SARS-CoV-2 M^pro^ and PL^pro^^52^, and cruzain^53^ share a similar enzyme reaction mechanism, we reasoned that inhibitors designed to target cruzain might be useful starting points for the development of SARS-CoV-2 protease inhibitors. Accordingly, we screened SARS-CoV-2 M^pro^ and PL^pro^ with the remnants of this collection that comprised 141 peptidic and peptidomimetic inhibitors^47^^,^^49^^,^^51^.

In the first-pass screens, none of the 141 compounds inhibited PL^pro^, whereas five compounds inhibited M^pro^. The two most potent inhibitors, termed **1a** and **5a**, were resynthesized for confirmation of inhibition. Also, the (R,S) enantiomer of **5a**, termed **5b**, was synthesised and found to inhibit the target. In concentration-response assays, all three compounds were nanomolar inhibitors of the M^pro^, as well as the related enzymes from SARS-CoV and MERS-CoV. Time-dependent assays of **1a**, **5a** and **5b** with SARS-CoV-2 M^pro^, supported by covalent and noncovalent docking and molecular dynamics (MD) simulations, indicated covalent inhibition. Also, rapid dilution enzymatic assays, supported by density functional theory (DFT) calculations, identified reversible inhibition for the three compounds. Last, characterisation of the binding site combined with docking analysis suggest that **5a** and **5b** are selective inhibitors of M^pro^ enzymes, with **5b** displaying the lowest inhibition values against SARS-CoV-2 M^pro^.

## Materials and methods

All mandatory laboratory health and safety procedures were complied with when performing experimental validation assays in this study, following international guidelines and our institutional safety protocols. Handling of chemical reagents and compounds and enzyme assays were performed with appropriate caution to minimise potential hazards and through the proper use of personal protective equipment (PPE). Cell-based assays require a Biosafety Level 2 (BSL-2) laboratory and were performed in the appropriate infrastructure following specific procedures and proper use of PPE. All experimental waste was appropriately segregated and disposed of according to institutional and regulatory guidelines. Relevant safety data sheets (SDS) and institutional guidelines should be consulted before replicating the experimental validation assays described herein.

### Collection of compounds

The collection of 141 compounds designed to target cruzain^47^^,^^49^^,^^51^ contains mainly dipeptidyl molecules with various reactive groups, such as vinyl sulphones, nitriles, ketones, epoxyketones, and acrylamides, with molecular weights ranging between 285.3 and 677.8 Da. All compounds were tested at the Center for Discovery and Innovation in Parasitic Diseases (CDIPD) in the Skaggs School of Pharmacy and Pharmaceutical Sciences (UCSD, USA). The list of all compounds in the legacy collection is available as supplementary information. The compounds’ nomenclature incorporates the acronym “WRR” after William R. Roush (of the former Scripps Research Institute, Jupiter, FL), the principal chemist involved in the previous studies. Compound powders were dissolved as 10 mM stocks in DMSO in 96-well polypropylene microplates, which were then stored at −80 °C.

### Expression and purification steps for recombinant SARS-CoV-2 M^pro^

Recombinant SARS-CoV-2 M^pro^ was expressed using a plasmid kindly provided by Dr. Rolf Hilgenfeld^54^ and purified following established protocols^43^^,^^54^ with minor adjustments. Briefly, the expression construct was transformed into *Escherichia coli* BL21-Gold (DE3) cells (Thermo Fisher Scientific, USA), which was then inoculated in 6 L of Luria Bertani (LB) broth (Thermo Fisher Scientific, USA) and incubated for 5 h at 37 °C. Cells were harvested by centrifugation (3000 × *g*, 15 min, 4 °C) and the cell pellet was resuspended in 30 mL of Buffer A (20 mM Tris-HCl, 150 mM NaCl, 0.1 mM DTT, and 5% glycerol, at pH 7.8) and lysed via microfluidization. Centrifugation (25 000 × *g*, 20 min, 4 °C) clarified the lysate, and the supernatant was applied to a HisTrap FF column (GE HealthCare, USA) equilibrated in Buffer A. After washing with 150 mL Buffer A to remove non-specific binders, M^pro^ was eluted with a 0 to 500 mM linear imidazole gradient over 30 column volumes using Buffer B (20 mM Tris-HCl, 150 mM NaCl, 1 mM DTT, 5% glycerol, and 500 mM imidazole, at pH 7.8). Pooled M^pro^-containing fractions were buffer-exchanged into Buffer A using 10 kDa MWCO centrifugal filters (Amicon Ultra, Merck, Germany). The His-tag was cleaved by adding 250 IU PreScission protease and incubating overnight at 4 °C. To remove the protease and tags, the sample was passed through tandem GSTrap FF and HisTrap FF columns (GE HealthCare, USA). The flow-through, containing the tag-free M^pro^, was concentrated and loaded into a HisTrap Q FF column (GE HealthCare, USA) equilibrated with Buffer C (20 mM Tris-HCl, 1 mM DTT, and 5% glycerol, at pH 8.0). Elution occurred via a 0 to 500 mM linear NaCl gradient over 20 column volumes using Buffer D (20 mM Tris-HCl, 1 M NaCl, 1 mM DTT, and 5% glycerol, at pH 8.0). A yield of 6.5 mg per litre of culture was obtained, and the final protein stock (37.96 μM) was stored in 20 mM Tris-HCl, 150 mM NaCl, 1 mM DTT, and 5% glycerol, at pH 8.0.

### Enzymes for inhibition assays

The recombinant SARS-CoV-2 M^pro^ was obtained as described above. The recombinant M^pro^ enzymes of SARS-CoV and MERS-CoV were purchased from R&D Systems (E720 and E719, respectively). Recombinant SARS-CoV-2 PL^pro^ was purchased from Acro Biosystems (PAE-C5184). Recombinant hCatL was purchased from R&D Systems (952-CY-010).

### SARS-CoV-2, SARS-CoV, and MERS-CoV M^pro^ inhibition assays

The inhibitor collection was screened at 10 µM against SARS-CoV-2 M^pro^ using a described assay.^55^ Briefly, assays were performed in 384-well black microplates following a 15-min pre-incubation of compounds with 100 nM enzyme diluted in 50 mM HEPES, 150 mM NaCl, 1 mM EDTA, and 0.01% Tween 20 at pH 7.5. The reaction was started by the addition of 20 μM of the fluorogenic substrate, Ac-Abu-Tle-Leu-Gln-MCA (Biosynth, FA178674) diluted with an equal volume of the same buffer (total volume of 30 μL). The final enzyme and substrate concentrations were 50 nM and 10 μM, respectively, and the final DMSO concentration was up to 0.2%. The substrate concentration in the assay was essentially twice the *K*_m_ value of 5.12 µM for M^pro^ (Supporting Information, Figure S1), allowing for the measurement of even a slight change in the enzyme’s binding efficiency in the presence of compound. Catalysis was monitored continuously for 2 h at 37 °C in a Synergy HTX (BioTek, Winooski, VT, USA) microplate reader with excitation/emission wavelengths of 360/460 nm. The M^pro^ inhibitor, nirmatrelvir (100 nM final concentration), was used as a positive control^56^. Enzyme activity was normalised to DMSO controls, and the percentage inhibition (mean ± standard error) was calculated. First-pass screens were performed as two independent assays, each in triplicate (*n* = 6 data points).

Compounds that inhibited enzyme activity by ≥ 50% progressed to eight-point concentration-response inhibition assays (0.00078 − 40 μM) of SARS-CoV-2 M^pro^, as well as the M^pro^ enzymes of SARS-CoV and MERS-CoV, as described^57^, following the same assay conditions as described above in the inhibitor collection screening. Briefly, 100 nM enzyme was diluted in the same buffer and incubated with eight-point concentrations of each compound in 384-well black microplates. After a 15-min pre-incubation, the reaction was started by the addition of 20 μM Ac-Abu-Tle-Leu-Gln-MCA diluted with an equal volume of the same buffer (total volume of 30 μL). Catalysis was monitored continuously for 2 h at 37 °C, as described above. Enzyme activity was normalised to DMSO controls, and the percentage inhibition (mean ± standard error)at each concentration was calculated. The half-maximal inhibitory concentration (IC_50_) was calculated by nonlinear regression (r^2^ > 0.9), and the deviation of each data point from the calculated nonlinear regression was < 10%. Two independent assays in triplicate were performed (*n* = 6 data points).

To evaluate time-dependent covalent inhibition, the same assay was performed without the 15-min pre-incubation step^58^. To measure reversible inhibition, SARS-CoV-2 M^pro^ was incubated at 100 times the final assay concentration for 30 min with the active inhibitors at 10-fold their respective IC_50_ values. A total of 0.5 μL of this mixture was transferred to 384-well black microplates and diluted 100-fold with assay buffer containing 10 μM Ac-Abu-Tle-Leu-Gln-MCA in a final volume of 50 μL. Thus, the final enzyme and inhibitor concentrations were 50 nM and 0.1-fold the IC_50_ value, respectively. Residual catalysis was monitored continuously for 2 h at 37 °C, as described above. Two independent assays in triplicate were performed (*n* = 6 data points). Data were analysed using GraphPad Prism 9.0 (GraphPad Software, San Diego, California, USA).

### SARS-CoV-2 PL^pro^ inhibition assays

Inhibition of recombinant PL^pro^ by each of the 141 compounds was measured using a previously described assay^52^^,^^59^. Briefly, 10 μM compound was pre-incubated for 15 min with 100 nM of enzyme in 50 mM HEPES, 150 mM NaCl, 0.01% Tween 20 and 0.1 mM dithiothreitol (DTT) at pH 6.5. Assays were performed in 384-well black microplates. The reaction was started by adding 100 µM of the fluorogenic substrate, Z-Arg-Leu-Arg-Gly-Gly-AMC (Bachem, 369 I1690), diluted with an equal volume of the same buffer (total volume of 30 μL). Catalysis was monitored continuously for 1 h at 37 °C, as described above. The final enzyme and substrate concentrations were 50 nM and 50 μM, respectively. GRL-0617 (10 μM final concentration) was used as a positive control^42^. Enzymatic activity was normalised to DMSO controls, and the percentage inhibition was calculated. One assay in triplicate was performed (*n* = 3 data points). Data were analysed using GraphPad Prism 9.0.

### hCatL inhibition assays

To understand whether another important cysteine protease for the SARS-CoV-2 cycle^37^, namely hCatL, could be inhibited, each of the hits identified from the M^pro^ assays was measured using a previously described assay^43^. Briefly, compounds were pre-incubated at 20 μM for 15 min with 160 pM of hCatL in 40 mM sodium acetate, 100 mM NaCl, 5 mM DTT, 1 mM EDTA and 0.001% bovine serum albumin (BSA) at pH 5.5. Assays were performed in 384-well black microplates using 40 μM of the fluorogenic substrate, Z-Phe-Arg-AMC (Bachem, I-1160), diluted with an equal volume of the same buffer (total volume of 30 μL). Catalysis was monitored continuously for 1 h at 25 °C, as described above. The final enzyme, substrate, and compound concentrations were 40 pM, 10 μM, and 10 μM, respectively. The cysteine protease inhibitor, E-64 (Research Products International, IL, USA), was used as a positive control (100 nM final concentration). Two independent assays in triplicate were performed (*n* = 6 data points).

Compounds that inhibited hCatL activity by ≥ 50% progressed to eight-point concentration-response inhibition assays (0.0000195 − 80 μM). K11777, which is currently being clinically assessed as a candidate to treat SARS-CoV-2 infections via its inhibition of hCatL^43^, was used as a positive control. The IC_50_ was calculated by nonlinear regression (r^2^ > 0.9), and the deviation of each data point from the calculated nonlinear regression was < 10%. Two independent assays in triplicate were performed (*n* = 6 data points). Data were analysed using GraphPad Prism 9.0.

### (Re)synthesis of compounds 1a, 5a and 5b

**Scheme 1.**
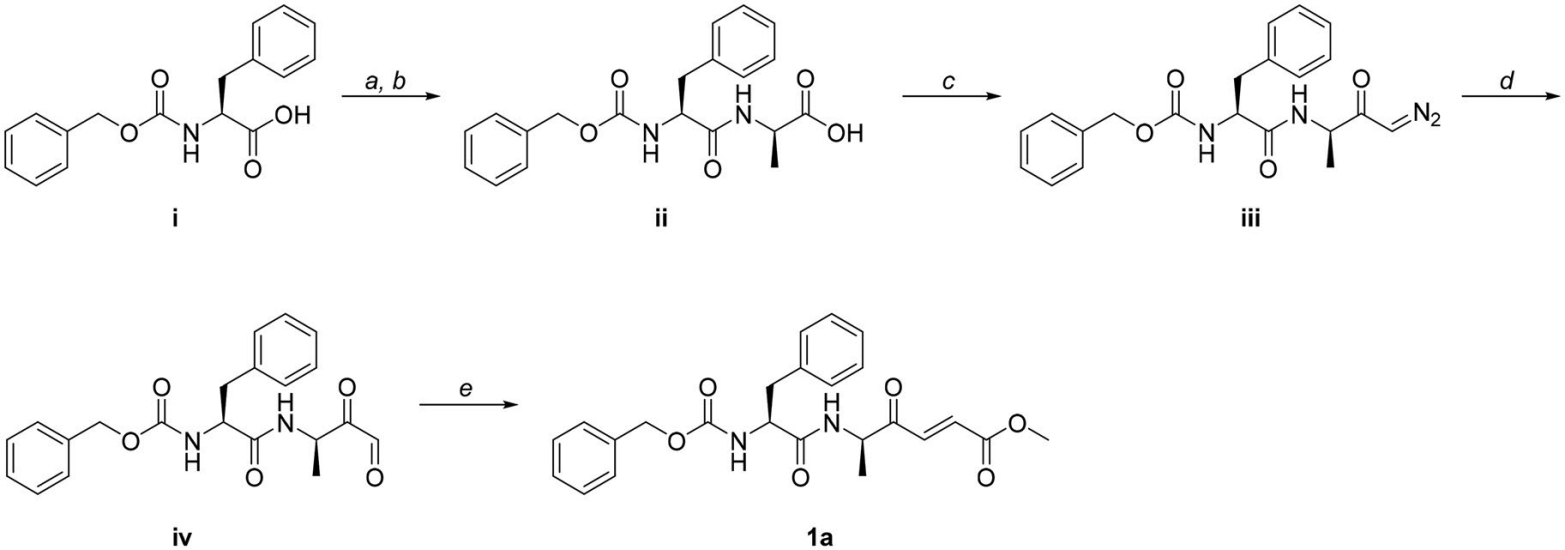
Reactions and conditions. (a) *N-*Cbz-*L*-phenylalanine **i** (1.00 eq), NHS (1.00 eq), DCC (1.10 eq), CHCl_3_ (0.05 M), room temperature (rt), 1 h. (b) *D*-alanine (1.00 eq), NaHCO_3_ (2.00 eq), H_2_O/acetone (1:2, 0.05 M), rt, 16 h (89% over two steps). (c) *N-*Cbz-*L*-phenylalanyl-*D*-alanine **ii** (1.00 eq), *N*,*O*-dimethyl-hydroxylamine hydrochloride (1.10 eq), ethyl chloroformate (1.00 eq), *N-*methylmorpholine (1.00 eq), freshly prepared diazomethane (6.00 eq), THF (0.1 M), −78 °C to rt, 16 h (37%). (d) Diazoketone **iii** (1.00 eq), freshly prepared DMDO (3.00 eq), acetone (0.02 M), −78 °C to rt, 16 h (99%). (e) Glyoxal **iv** (1.00 eq), methyl 2-(dimethoxyphosphoryl)acetate (1.10 eq), NaH (1.10 eq), CH_3_CN (0.05 M), 0 °C to rt, 16 h (12%).

Compound **1a** was synthesised in several steps following methods previously reported^60^^,^^61^, with some modifications (Scheme 1). *N-*Cbz-*L*-phenylalanyl-*D*-alanine **iii** was synthesised via DCC-mediated peptide coupling of *N*-Cbz-*L*-phenylalanine **i** with *D-*alanine **ii**. The Weinreb amide of **ii** was synthesised via reaction with *N*,*O*-dimethyl-hydroxylamine hydrochloride and mixed anhydride intermediate, which was subsequently reacted with freshly prepared diazomethane to furnish the diazoketone **iii** intermediate. Glyoxal **iv** was synthesised via oxidative cleavage of **iii** using freshly prepared dimethyldioxirane, followed by condensation with the appropriate ylide to furnish inhibitor **1a**.

**Scheme 2.**
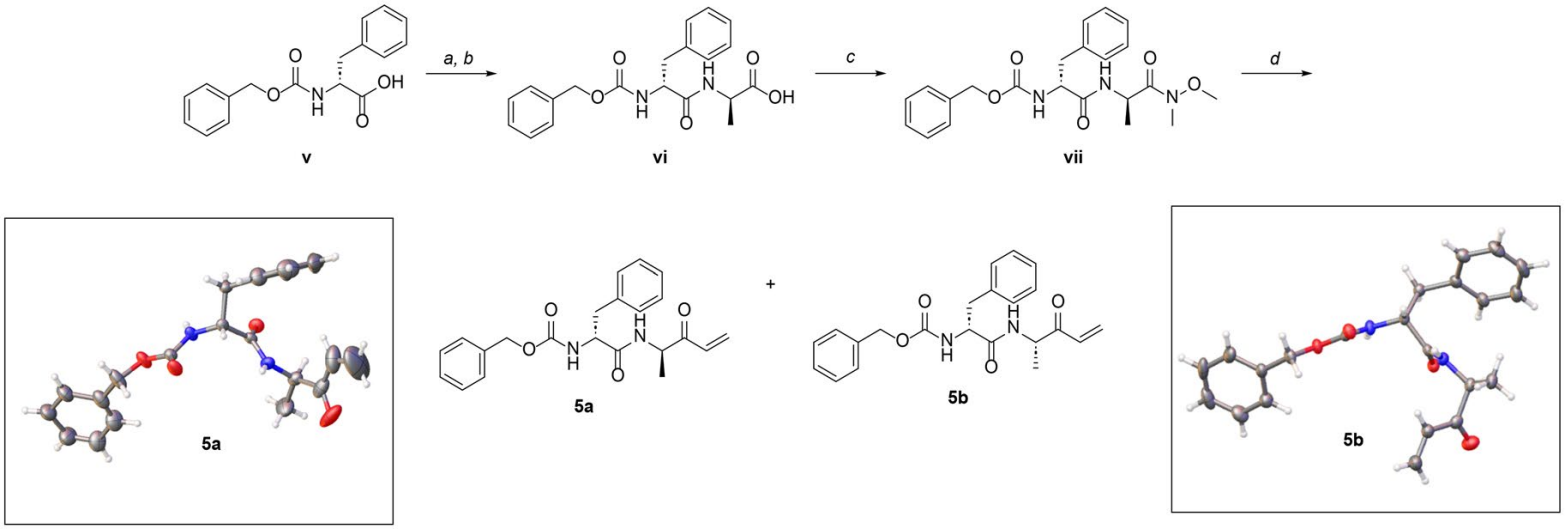
Reactions and conditions. (a) *N-*Cbz-*D*-phenylalanine **v** (1.00 eq), NHS (1.00 eq), DCC (1.10 eq), CHCl_3_ (0.05 M), rt, 1 h. (b) *D*-alanine (1.00 eq), NaHCO_3_ (2.00 eq), H_2_O/acetone (1:2, 0.05 M), rt, 16 h (91% over two steps). (c) *N-*Cbz-*D*-phenylalanyl-*D*-alanine **vi** (1.00 eq), *N,O-*dimethyl-hydroxylamine hydrochloride (1.10 eq), HATU (1.10 eq), DIPEA (2.50 eq), DMF (0.1 M), rt, 16 h (88%). (d) Weinreb amide **vii** (1.00 eq), vinyl magnesium bromide (3.00 eq), THF (0.1 M), 0 °C to rt, 3 h (58%).

Compounds **5a** and **5b** were synthesised in parallel starting from the *N-*Cbz-*D*-phenylalanine **v** (Scheme 2). *N-*Cbz-*D*-phenylalanyl-*D*-alanine **vii** was synthesised via DCC-mediated coupling of **v** with *D*-alanine, followed by HATU-mediated formation of Weinreb amide **vii**. Amide **vii** was subjected to Grignard reaction using vinyl magnesium bromide, which provided diastereomers **5a** and **5b** at equimolar ratios. Compounds **5a** and **5b** were separated by chiral HPLC, and their absolute stereochemistry was assigned by single crystal X-ray diffraction. Chemistry for the synthesis of compounds **ii**, **iii**, **iv**, **1a**, **vi**, **vii**, **5a,** and **5b** (Schemes 1 and 2) and single crystal structure reports for compounds **5a** and **5b** (Tables A–E) are available in the Supplemental Material.

### Cytotoxicity evaluation

The concentration at which the viability of Vero (green monkey kidney fibroblast; ATCC^®^ CCL-81^™^) and L929 (mouse subcutaneous adipose fibroblasts; ATCC^®^ CCL-1^™^) cells was reduced by 50% (CC_50_ value), was calculated using the MTT (3–(4,5-dimethylthiazol-2-yl)-2,5-diphenyltetrazolium bromide; Thermo Fisher Scientific, USA) assay^62^. Cells were cultured in DMEM (Cultilab, Brazil) supplemented with 5% heat-inactivated foetal bovine serum (FBS) (Cultilab, Brazil), 100 IU/mL penicillin (Cellofarm, Brazil), 100 μg/mL streptomycin (Merck, Germany) and 0.25 μg/mL amphotericin B (Cultilab, Brazil) at 37 °C and 5% CO_2_. To perform the assays, cells were seeded into 96-well microplates at 4 x 10^4^ cells/well in 100 μL DMEM with 1% FBS and incubated for 24 h. The medium was then replaced with 200 μL medium containing seven-point serial dilutions of compound (200 − 3.125 μM) and incubated for 48 h. After incubation, the medium was removed, and 100 μL MTT in DMEM containing 1% FBS (0.5 mg/mL) were added to each well and incubated. After 3 h, the medium was removed and 100 μL DMSO added to solubilise the formazan crystals, followed by shaking the microplates using a magnetic stirrer for 20 min. Measurement of dissolved formazan was performed using a spectrophotometer (VersaMax, San Jose, CA, USA) at 570 nm, as described^63^. Growth inhibition (%) at each compound concentration was normalised to DMSO controls (0.78 to 0.006% v/v). Linear regression was used to calculate CC_50_ values and considering only those data for which r^2^ > 0.85. Two independent assays in triplicate were performed (*n* = 6 data points). Data were analysed using GraphPad Prism 9.0.

### Binding site characterisation of SARS-related and MERS-CoV M^pro^

The SARS-CoV-2 M^pro^ structure co-crystallized with GC376 (the bisulphite adduct of GC373), a reversible and covalent cysteine protease inhibitor of feline infectious peritonitis virus (FIPV) among other coronaviruses^64^, was selected from the Protein Data Bank^65^ (PDB ID: 7D1M^66^) due to its high 1.35 Å resolution and the relevance of the binding ligand^67^. In addition, the SARS-CoV M^pro^ (PDB ID: 6W2A^68^, 1.65 Å resolution) and MERS-CoV M^pro^ (PDB ID: 6VH3^68^, 2.2 Å resolution), both co-crystallized with the inhibitor, **7j**^68^ (IUPAC name (1S,2S)-2-[[(2S)-2-[(4,4-difluorocyclohexyl)methoxycarbonylamino]-4-methylpentanoyl]amino]-1-hydroxy-3-[(3S)-2-oxopyrrolidin-3-yl]propane-1-sulfonic acid)), were included for comparison. The structures were submitted to the web servers, FTSite^69^ and PrankWeb^70^. FTSite predicts binding sites and amino acid residues for possible ligand interactions, and PrankWeb predicts and ranks the potential binding points clustered on the protease surface by calculating the physicochemical and geometric properties of residues. Thus, only residues predicted by the two different web servers are considered relevant and selected in a consensus prediction. The PyMOL^71^ software (v2.5.7 Schrödinger, Inc., New York, NY, USA) was used to generate images.

### Preparation of compounds for docking and MD simulations

Ligands were prepared as previously reported^72^. Briefly, 3D structural models of the compounds were generated using Discovery Studio (BIOVIA, USA, 2017). The compounds were then submitted to OMEGA 3.1.1.2 (OpenEye Scientific Software, USA, 2019) to create a conformer set^73^. The lowest energy of each compound was generated using the command line “–maxconfs 1” i.e. the most stable conformational unit of a compound was generated among a maximum number of possible conformations. All structures had their ionisation states corrected to pH 7.4 employing the FixpKa application in the QUACPAC 2.0.1.2 software (OpenEye Scientific Software, USA, 2019) to allow for a single favourable ionisation state.

### Noncovalent molecular docking of compounds with SARS-related and MERS-CoV M^pro^

Noncovalent molecular docking of the SARS-CoV-2, SARS-CoV and MERS-CoV M^pro^ structures were performed with the GOLD 5.1^74^ software. For comparison, we also performed docking of hCatL (PDB ID: 2XU3^75^, 0.90 Å resolution) with the nitrile inhibitor, **XU3** (IUPAC name (2S,4R)-4–(2-chlorophenyl)sulfonyl-1-[1–(5-chlorothiophen-2-yl)cyclopropyl]carbonyl-N-[1-(iminomethyl)cyclopropyl]pyrrolidine-2-carboxamide). In addition, docking of SARS-CoV-2 M^pro^ was performed with Glide^76^ to be used as an input for MD simulations. The intrinsic parameters of the programs, such as scoring function, the flexibility of residues and atoms and the number of poses, i.e. predicted binding modes, were standardised to better reproduce the experimental binding mode of co-crystallized ligands, as previously described^77^. The redocking of the co-crystallized ligands considered root-mean-square deviation (RMSD) values ≤ 2.0 Å^78^. The redocking poses provided binding mode references for comparison purposes, while also validating the docking protocol. Target structures were prepared by fixing missing sidechains, adding hydrogen atoms, removing water molecules, and calculating atomic partial charges using the default parameters^79^. The search parameters in GOLD were: (i) all protein rotatable bonds were fixed; (ii) binding site defined with the co-crystallized ligand with all atoms within 6 Å; (iii) chemscore_kinase as the template; (iv) 200 genetic algorithm (GA) runs; (v) CHEMPLP as the scoring function, with no early termination allowed; and (vi) the GA search option set as slow. The PyMOL software (v2.5.7) was used to generate images.

### Covalent molecular docking and pose selection of compounds with SARS-CoV-2 M^pro^

Covalent docking of the SARS-CoV-2 M^pro^ structure was performed using Glide^76^ v7.7 with extra precision (XP) mode, as described^80^. Briefly, the selected compounds were prepared using LigPrep (Schrödinger, LLC, New York, NY, 2024) to assign their protonation state (at pH 7.0 ± 1.0) and partial charges. First, a noncovalent docking was predicted considering a pocket of 13 Å around the co-crystallized ligand GC376 (PDB ID: 7D1M). These generated up to 10 predicted poses per compound that were used for covalent docking using CovDocking. The predicted covalent docking poses were generated with similar parameters by selecting a hydrothiolation (thiol-ene) reaction with the catalytic Cys145 as the reactive residue. Representative poses were selected based on the docking score and by visual inspection of the warhead position covalently bound to the cysteine. The PyMOL software (v2.5.7) was used to generate images.

### MD simulations and trajectory analyses of SARS-CoV-2 M^pro^ inhibitors

MD simulations were performed as described^57^^,^^81^ to analyse the stability of the SARS-CoV-2 M^pro^ enzyme-inhibitor complexes. Briefly, all simulations were conducted using the Desmond software^82^ and OPLS4 force field^83^ for the protein and ligand parameters. The systems were solvated with the TIP3P explicit solvent model^84^ in a cubic periodic box. In addition, counter ions were added to neutralise the overall system charge (e.g. Na^+^ or Cl^−^ depending on system). The dimensions of the simulation cubic box were set with periodic boundary conditions equal to 13 Å. The long-range electrostatic interactions were calculated using the Smooth Particle Mesh Ewald (PME) method, while short-range interactions were calculated using 1 fs time steps and considering a 9 Å cut-off value. These parameters are sufficient to approximate long-range interactions on large timescales^85^^,^^86^. A constant temperature of 310 K was maintained using the Nose-Hoover thermostat algorithm,^87^ and a constant pressure of 1 atm was maintained using the Martyna-Tobias-Klein Barostat algorithm^88^^,^^89^. The system was minimised and relaxed, followed by a multiple production step of at least 200 ns, having frames being recorded and saved every 1000 ps.

Simulations were carried out to a total of 2 µs per ligand (10 independent replicates of 200 ns) using random seeds. The trajectories and interaction data are available on the Zenodo repository under the code: 10.5281/zenodo.7841336. Representative structures were selected using hierarchical clustering analysis (HCA) of the equilibrated trajectories based on changes in the RMSD, i.e. a representative frame was selected at random at points along the trajectory when the RMSD was not fluctuating after equilibration. Protein-ligand interactions were analysed using the Simulation Interaction Diagram pipeline in Maestro (Maestro v2023.1) with a cut-off of 10% for predicted interactions. Lastly, the binding energies of the ligands were calculated using the molecular mechanics-generalized Born and surface area continuum solvation (MM/GBSA) models as implemented in Prime^90^ (thermal MM/GBSA script). The calculated MM/GBSA data was used to represent free binding energies, being normalised by the number of heavy atoms (HAC) according to the following formula: ligand efficiency = ln (docking score)/(1 + ln (HAC)).

### DFT calculations

The most potent compounds, **5a** and **5b**, were submitted to DFT calculations and compared to M^pro^ inhibitor controls, i.e. nirmatrelvir as a reversible inhibitor, and GC373 as an irreversible inhibitor^64^. This method aims to calculate the energy of adduct formation with a given cysteine residue, for example, the catalytic Cys145 of SARS-CoV-2 M^pro^, as previously reported^91^. Briefly, we used the B3LYP functional^92^^,^^93^ with 6–31 G* and 6–311++G** basis sets^94^^,^^95^ performed using a geometric direct minimisation to an implicit solvation model with water. The set calculations were performed in Spartan ‘20 (v. 1.1.4, Wavefunction Inc., CA, USA) using the C-PCM method^96^ and considering a dielectric of 78.30. Calculations were performed using the 3D structure of inhibitors building the cysteine and cysteine-compound adducts as previously described^97^. Last, the energy of adduct formation, i.e. the energy of binding, was calculated as the energy of the adduct minus the sum of the ligand energy and the cysteine energy.

## Results

### M^pro^ inhibitors identified in a legacy collection of cysteine protease inhibitors

**Figure 1.**
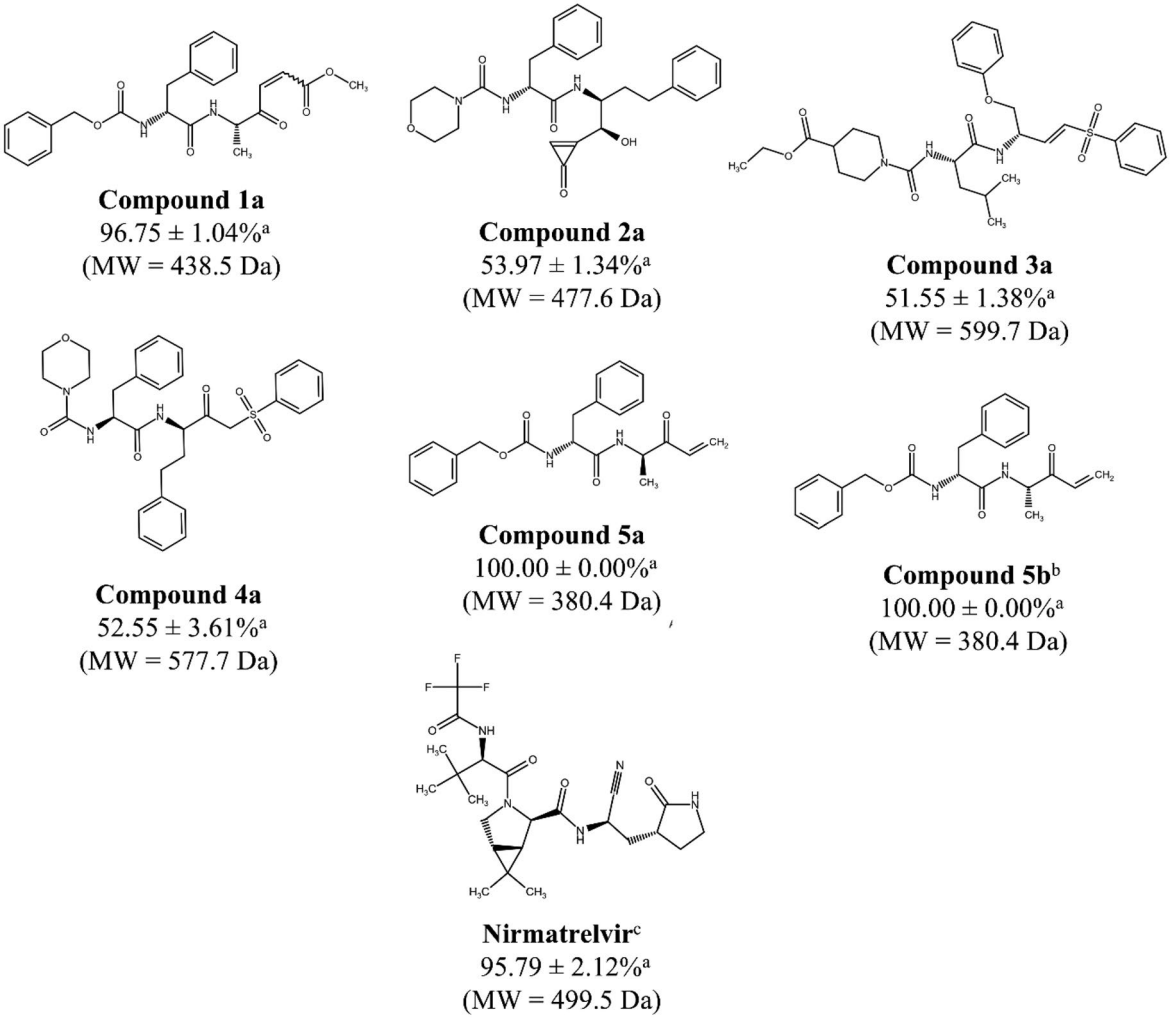
Compounds that inhibit SARS-CoV-2 M^pro^ activity at 10 μM. ^a^Percent inhibition relative to DMSO control is reported as the mean ± SE calculated from two independent assays in triplicate (*n* = 6). Errors are given by the ratio of the standard deviation to the square root of the number of measurements. MW corresponds to molecular weight. ^b^Compound **5b** was synthesised based on the activity observed for **5a**. ^c^The M^pro^ inhibitor, nirmatrelvir, used as a positive control, was tested at 100 nM.

From 141 compounds subjected to first-pass screens at 10 µM against SARS-CoV-2 M^pro^ (Table S1), five hits (**1a**–**5a**) were identified that reduced enzyme activity by ≥ 50%. Two compounds, **1a** and **5a**, inhibited enzyme activity by > 95%. Both possessed a benzyl carbamate group, with **1a** having an electrophilic ethene-ester warhead, and **5a**, an electrophilic vinyl ketone group (Figure 1). Against SARS-CoV-2 PL^pro^, none of the 141 compounds at 10 µM inhibited activity by ≥ 50% (Table S2).

Compounds **1a** and **5a**, the most potent inhibitors (Figure 1), were resynthesised because they had been stored for > 10 years. In addition, we synthesised the (R,S) enantiomer of **5a**, termed **5b**. The IC_50_ values of **1a** through **5a and 5b** against SARS-CoV-2 M^pro^ ranged from 0.128 to 16.42 μM (Table 1 and Figure S2). Compounds **2a** through **4a** were weakly active (∼8 to 16 μM), whereas **1a** and **5a** were active in the submicromolar range, at 0.415 and 0.1601 μM, respectively. Compound **5b** was the most potent inhibitor of SARS-CoV-2 M^pro^ with an IC_50_ value of 0.128 μM, approximately 7-fold less potent than nirmatrelvir’s IC_50_ of 0.018 μM. Among the six hit compounds, only **1a**, **5a,** and **5b** were active against SARS-CoV M^pro^ with **1a** and **5a** being active against MERS-CoV M^pro^ (Table 1 and Figure S3). Compound **5b** was also the most potent inhibitor against SARS-CoV M^pro^ with an IC_50_ value of 0.0732 μM, only 2.4-fold greater than the value for nirmatrelvir (0.0302 μM). Against hCatL, **1a** through **3a** were active in the submicromolar range (0.184 to 0.763 μM), whereas **4a** was weakly active, with an IC_50_ value of 10.74 μM (Table 1 and Figure S2). Conversely, **5a** and **5b**, the most potent M^pro^ inhibitors, were inactive against hCatL (Table 1).

**Table 1.** IC_50_ values of hit compounds against different coronavirus M^pro^ and hCatL.

Compound	IC_50_ (μM)			
	SARS-CoV-2 M^pro^	SARS-CoV M^pro^	MERS-CoV M^pro^	hCatL
**1a** ^a^	0.415 ± 0.018	0.6995 ± 0.0125	3.786 ± 0.640	0.319 ± 0.001
**2a**	8.12 ± 0.04	>10	>10	0.184 ± 0.003
**3a**	16.42 ± 0.09	>10	>10	0.763 ± 0.079
**4a**	8.72 ± 0.54	>10	>10	10.74 ± 0.24
**5a** ^a^	0.1601 ± 0.0058	0.1105 ± 0.0173	0.8295 ± 0.0535	>10
**5b** ^a^	0.128 ± 0.006	0.0732 ± 0.0054	NT	>10
**Nirmatrelvir**	0.0181 ± 0.0005	0.0302 ± 0.0024	0.0881 ± 0.0074	NT
**K11777**	NT	NT	NT	0.000614 ± 0.000082
**E-64** ^b^	NT	NT	NT	0.008 ± 0.001

Two independent assays performed in triplicate (*n* = 6 data points).

^a^ (Re)Synthesised compounds.

^b^ E-64 data were reported previously^91^.

NT: not tested.

### The predicted interactions of compounds 5a and 5b support their potency and specificity for M^pro^

To understand the target selectivity of the M^pro^ inhibitors, **5a** and **5b**, we focused on characterising the amino acid residues conserved between the M^pro^ enzymes of SARS-CoV-2, SARS-CoV and MERS-CoV. Characterisation of the binding sites was conducted using FTSite, and 11 residues (Thr26, Leu27, His41, Phe140, Leu141, Gly143, Ser144, Cys145, His163, Met165 and Glu166) were identified for the three enzymes (Figure S4). In addition to these, two important substitutions were noted between the two SARS-related enzymes and MERS-CoV M^pro^, namely Asn142Cys and His164Gln (Figure S4D), which might influence potential inhibitor interactions. In addition, His172 of MERS-CoV, Met49 of SARS-CoV-2, and Thr25 of SARS-CoV-2 and SARS-CoV M^pro^ were also predicted to interact. All these residues (and their substitutions) were also predicted by PrankWeb to be favourable for ligand interaction (Table S3), thus corroborating the FTSite results (Figure S4).

**Figure 2.**
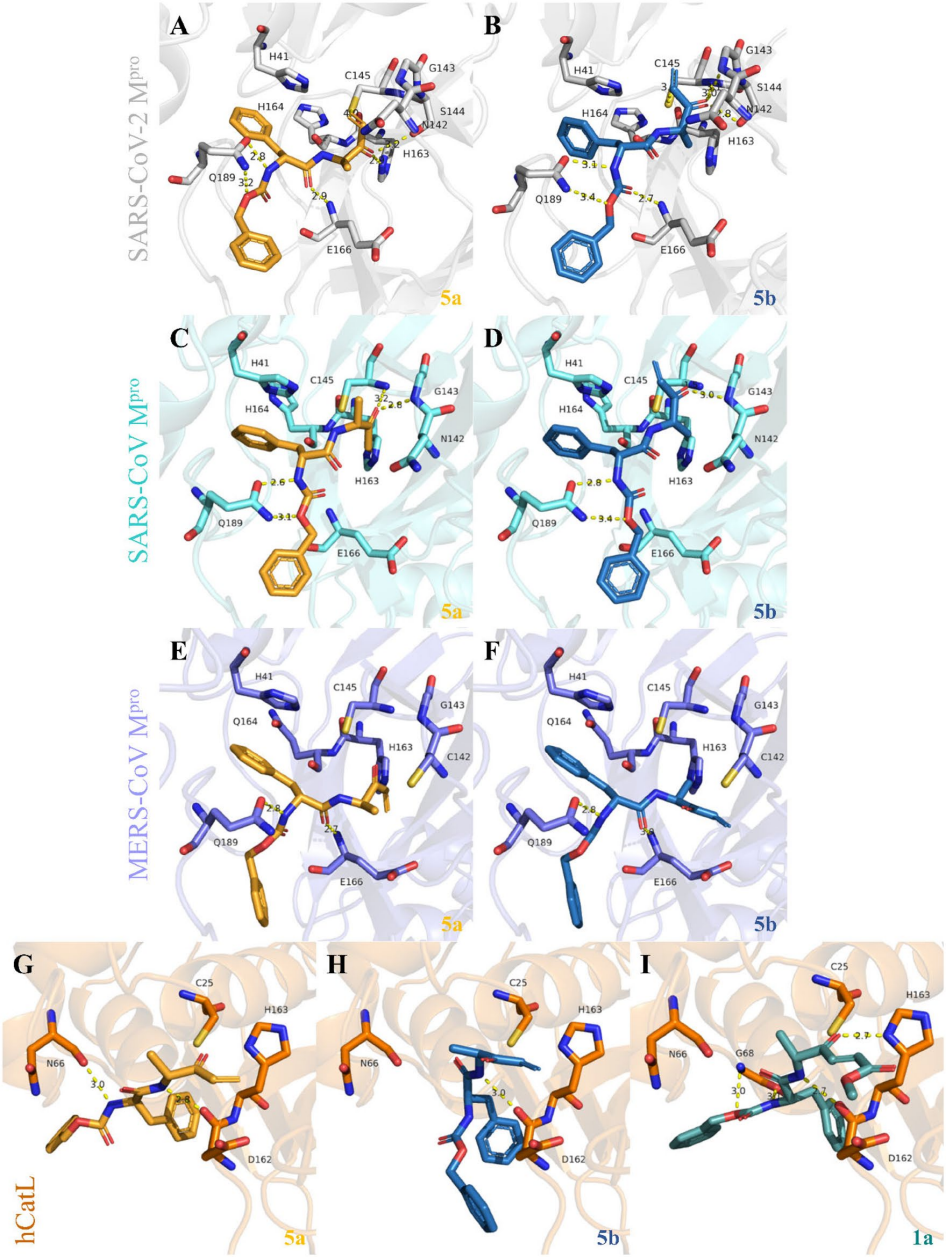
Proposed binding modes of **5a** and **5b** with different cysteine proteases. Noncovalent docking was performed using GOLD. Similar binding poses were predicted for both **5a** (bright orange) and **5b** (sky blue) with SARS-CoV-2 M^pro^ (grey, A and B, respectively) and SARS-CoV M^pro^ (aquamarine, C and D, respectively). In contrast, the predicted interactions of **5a** (E) and **5b** (F) with MERS-CoV M^pro^ (slate blue) show that the electrophilic vinyl ketone warhead faces away from the catalytic cysteine, despite having other hydrogen bond interactions in the site. The warhead for **5a** (G) and **5b** (H) is also predicted to face away from the catalytic Cys25 residue of hCatL (orange), which supports their absence of activity against this enzyme *in vitro*. Conversely, compound **1a** (I, light teal) is predicted to interact with the catalytic His163 and other residues in the active site of hCatL. The catalytic residues, His41 and Cys145 (M^pro^), and Cys25 and His163 (hCatL), are displayed as sticks and labelled black, along with other important residues for these enzymes. Hydrogen atoms are hidden for clarity. The predicted interactions and measured distances (vinyl ketone with Cys145) are shown as yellow dashed lines. Images were generated with PyMOL (v2.5.7).

Next, to understand whether these residue substitutions would impact inhibitor binding, we performed noncovalent docking using GOLD (Figure 2) for the most potent inhibitors, **5a** and **5b**, with the three coronavirus M^pro^ enzymes and hCatL. The method was validated with the redocking of each enzyme’s co-crystallized ligand (RMSD values ≤ 2.0 Å^78^) resulting in RMSD values of 1.92, 0.73 and 1.49 Å for SARS-CoV-2, SARS-CoV and MERS-CoV M^pro^, respectively, and 0.84 Å for hCatL (Figure S5). Our redocking results for SARS-CoV-2 M^pro^ match those found in the original docking study with GC376, which used GLIDE as the software^66^. Cross-studies and software validation are important to accurately predict residues that interact with a ligand and its overall pose, which was the case in the design of GC376 against SARS-CoV-2 M^pro^^66^^,^^67^, SARS-CoV and MERS-CoV M^pro^^98^, and the Michael acceptor inhibitor, **N3**, against the same enzymes^41^.

Our noncovalent docking of **5a** and **5b** with SARS-CoV-2 M^pro^ predicted similar poses for both compounds (Figures 2A and 2B). Hydrogen bond interactions were predicted between the compounds’ P4 benzyl-ester groups and Gln189, the P3 amide linker and the backbone of Glu166, and the P1 ester with Ser144. The electrophilic group (vinyl ketone) of both **5a** and **5b** was predicted to face towards the catalytic cysteine, likely establishing a covalent reaction (distances measured were 4.0 and 3.6 Å, respectively). Compound **5b** was also predicted to have other P1 hydrogen interactions with both Cys145 and Gly143, whereas **5a** has just one predicted P1 contact with His163. The additional interactions of **5b** over **5a** correlate with their enzyme-inhibition activities (Table 1). Similar poses were predicted for **5a** and **5b** in SARS-CoV M^pro^, which maintained the Gln189 contacts, but had no predicted interactions with Glu166 (Figures 2C and 2D). In contrast, in MERS-CoV, **5a** and **5b** were predicted to have the P1 vinyl ketone group facing away from the catalytic cysteine (Figures 2E and 2F), with just one interaction with Gln189 and Glu166, thereby consistent with weaker binding overall, possibly due to the interference of the Asn142Cys and His164Gln substitutions (Figure S4D). In support of these predictions, **5a** inhibited MERS-CoV M^pro^ with a ∼5-fold decrease in activity compared to SARS-related M^pro^ enzymes (Table 1). Last, docking poses for **5a** and **5b** in hCatL also show that the P1 is predicted to face away from the catalytic Cys25 (Figures 2G and 2H), with just one interaction with either Asn66 or Asp162, consistent with both compounds’ lack of inhibition of hCatL (Table 1). In contrast, **1a**, which was active against hCatL, is predicted to interact with Asp162 and Gly68 in the active site of hCatL, as well as one additional contact with the catalytic His163 (Figure 2I).

### Compounds 1a, 5a and 5b are covalent reversible inhibitors of SARS-CoV-2 M^pro^

**Figure 3.**
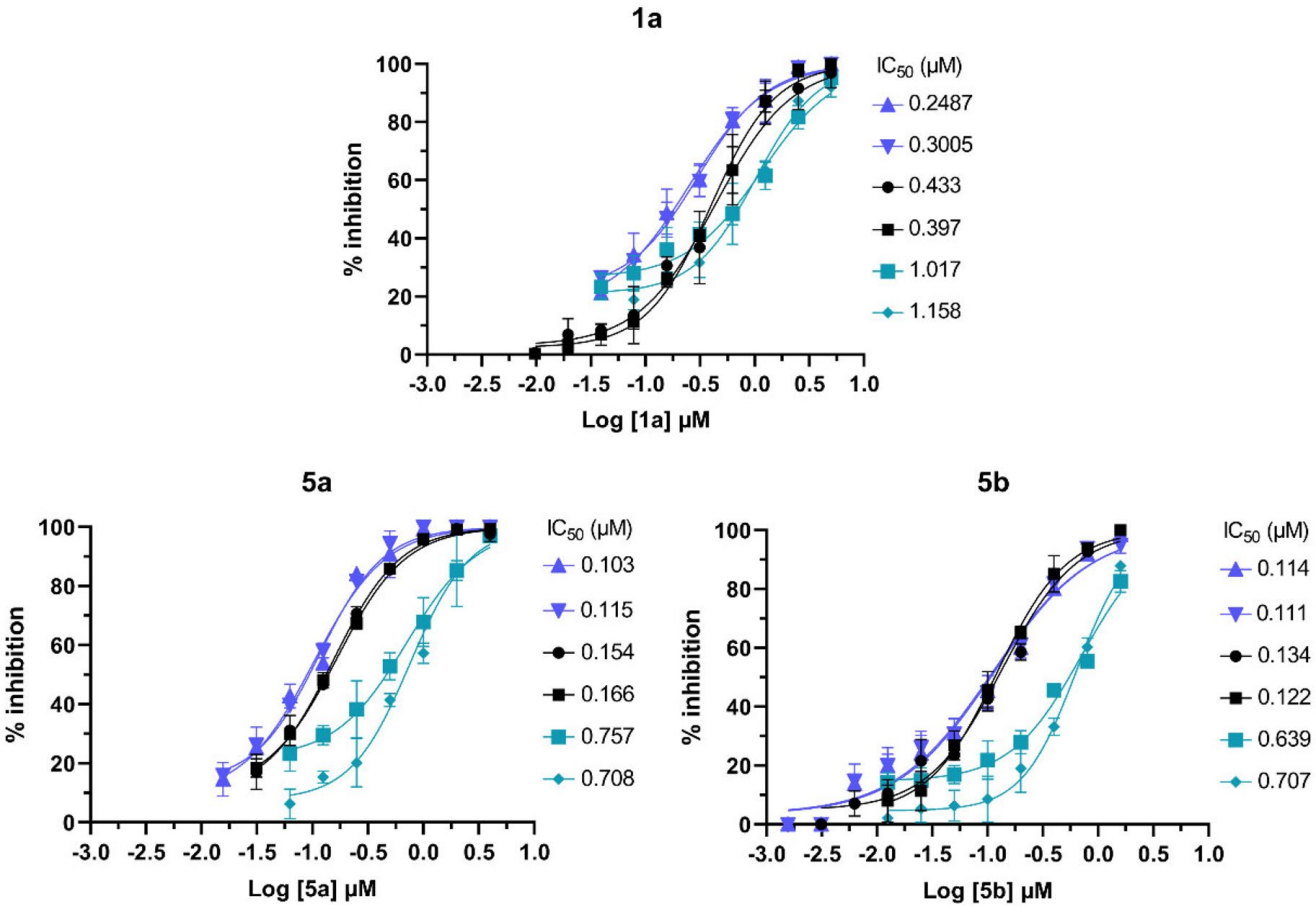
Time-dependent concentration-response curves for inhibition of SARS-CoV-2 M^pro^ activity by compounds **1a**, **5a,** and **5b**. Two independent assays performed in triplicate (*n* = 6 data points). The violet, black, and teal curves represent assay data generated after a 30-, 15-, or 0-min pre-incubation period, respectively, prior to addition of substrate.

Considering the likelihood of establishing a covalent reaction (distances measured of 4.0 and 3.6 Å, Figures 2A and 2B), we next asked whether the most potent SARS-CoV-2 M^pro^ inhibitors, **1a**, **5a** and **5b**, showed time-dependent inhibition, which would support covalent binding.^58^ We compared the inhibition-response curves obtained with and without a 15-min pre-incubation period before addition of substrate (Figure 3). The curves with pre-incubation were shifted to the left resulting in essentially 2-fold (compound **1a**) and 4-fold (**5a** and **5b**) decreases in the respective IC_50_ values. In other words, pre-incubation notably increased inhibitor potency. The shifts observed, therefore, support covalent behaviour. No further shift was observed for the three compounds after a 30-min pre-incubation, suggesting that the 15-min pre-incubation time is sufficient to complete the covalent reaction^99^ Nonetheless, it is possible that changes in protein conformation could result in changes in enzymatic inhibition activity^100^^,^^101^, such as an induced fit or a higher affinity binding, which may influence the proposed covalent reaction mechanism, as has been observed for dipeptidyl inhibitors with other cysteine proteases, like cruzain^102^.

**Figure 4.**
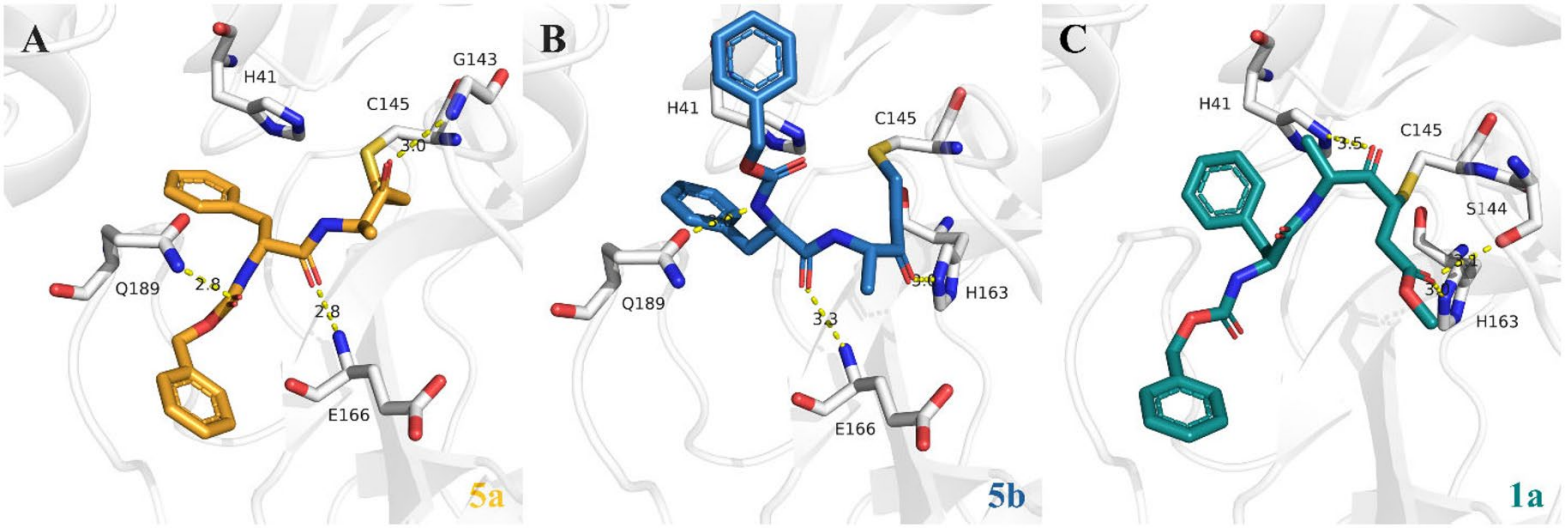
Proposed covalent binding modes of 5a, 5b, and 1a with SARS-CoV-2 M^pro^. Covalent docking was performed using Glide XP. Similar binding poses were predicted for **5a** (A, bright orange)**, 5b** (B, sky blue), and **1a** (C, light teal) with SARS-CoV-2 M^pro^. Both enantiomers are predicted to have P4 interactions with Gln189 and P3 interactions with Glu166. In addition, **5a** is predicted to have a P1 contact with Gly143, while **5b** interacts with His164. The benzyl carbamate, **1a**, is not predicted to have P3 or P4 interactions, having only one P2 contact with His41, and two P1 contacts with Ser144 and His163. The catalytic residues, His41 and Cys145, are displayed as sticks and labelled black, along with other important residues. Hydrogen atoms are hidden for clarity. The predicted interactions and measured distances are shown as yellow dashed lines. Images were generated with PyMOL (v2.5.7).

To understand this covalent binding, we also performed a covalent docking of the three compounds that share a benzyl carbamate moiety, **1a**, **5a**, and **5b**, with SARS-CoV-2 M^pro^ using Glide XP (Figure 4). The predicted pose of **5a** has its P4 amide linker interacting with Gln189, moving the benzyl-ester to face down in the pocket (Figure 4A), whereas for **5b** the P4 still interacts with Gln189 and moves the benzyl-ester to face upwards (Figure 4B). In addition, for both compounds, the P3 amide linker is predicted to interact with the backbone of Glu166. Similar interactions are also predicted in our noncovalent docking (Figures 2A and 2B). With the electrophilic group (vinyl ketone) now covalently bonded to Cys145, the P1 amide of **5a** is predicted to interact with Gly143 (Figure 4A), while **5b** interacts with His164 (Figure 4B), supporting the stabilisation of the covalent reaction poses. Conversely, the less potent benzyl carbamate, **1a**, is predicted to have different interactions (Figure 4C). Specifically, hydrogen bonds are predicted between the P2 amide linker and His41, and between the P1 amide linker and Ser144 and His163. However, because **1a** lacks the additional P3 and P4 interactions predicted for **5a** and **5b**, its overall binding to SARS-CoV-2 M^pro^ is likely to be weaker. These predicted inhibitor interactions also correlate with the differences in these three compounds’ enzyme-inhibition activities (Table 1) and the covalent inhibition indicated by the time-dependent assays (Figure 3).

**Figure 5.**
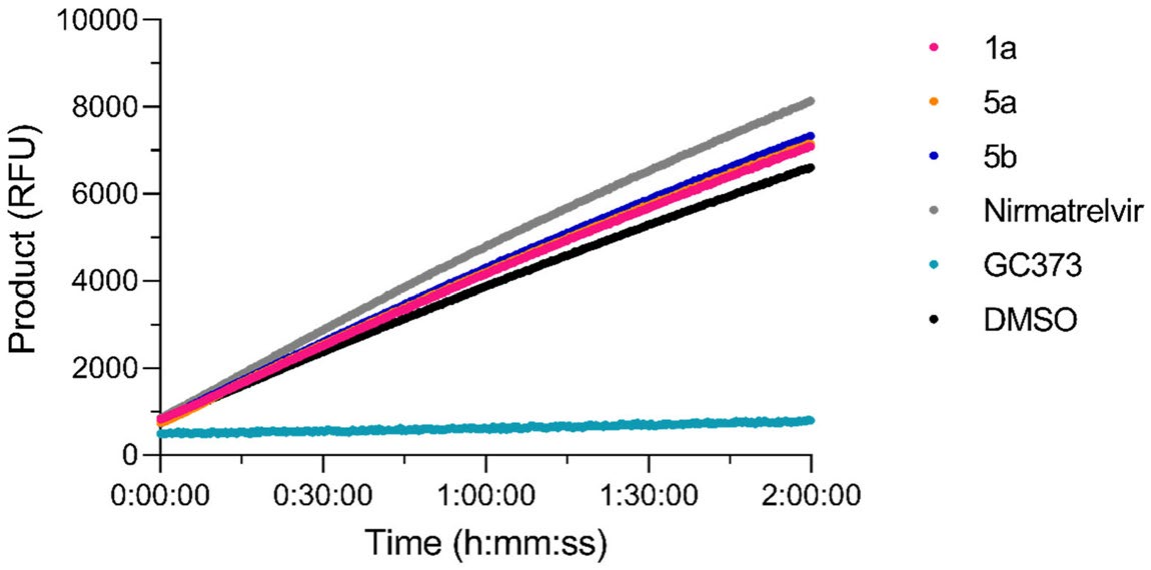
Inhibition of SARS-CoV-2 M^pro^ by compounds **1a**, **5a,** and **5b** is reversible. Following pre-incubation of the enzyme with excess inhibitor, the mixture was diluted, and the formation of the cleavage product was monitored for 2 h. Two independent assays in triplicate were performed (*n* = 6 data points). Image displays the average of three data points of product formation (i.e. cleaved substrate) represented by the relative fluorescence units (RFU) over time (h:mm:ss).

Rapid dilution assays were then conducted to investigate whether the indicated covalent inhibition of **1a**, **5a** and **5b** is reversible. These assays have been used before to evaluate the reversible-binding behaviour of covalent inhibitors^103^. Here, compounds were pre-incubated at 10-fold their IC_50_ values with 100× of the enzyme’s final assay concentration of 50 nM. The mixture was diluted to achieve the predetermined proportions of substrate and enzyme of 10 μM and 50 nM, respectively, and 0.1-times the given compound’s IC_50_ value. Nirmatrelvir and GC373 were used as reversible and irreversible inhibitor controls, respectively. After dilution, **1a**, **5a**, **5b,** and nirmatrelvir, did not maintain inhibition of the enzyme, i.e. reversible inhibition, whereas inhibition by GC373 was unaffected (Figure 5).

### MD simulations and DFT calculations support reversible inhibitor binding modes for compounds 5a and 5b with SARS-CoV-2 M^pro^

We next performed noncovalent MD simulations to assess the binding stability of the enantiomers, **5a** and **5b**, in SARS-CoV-2 M^pro^, as well as predict their frequency of interaction with the enzyme residues. Initially, we validated the system by simulating the co-crystallized cysteine protease inhibitor, GC376 (PDB ID: 7D1M). Five replicates of 200 ns, i.e. 1 µs, were simulated to predict the residues that bind to GC376 and their frequencies of interactions: Phe140 (48% over the analysed trajectory), Cys145 (93%), His163 (90%), His164 (75%), Glu166 (96%) and Gln189 (74%), as well as with Thr26 (30%) and Gly143 (40%), which are mediated by water contacts (Figure S6A). Cys145 was predicted to interact with the P1 hydroxyl group of GC376, whereas Phe140, His163, His164, Glu166 and Gln189 were predicted to interact with the amide. Simulations with GC376 co-crystallized with a monomer of SARS-CoV-2 M^pro^ (PDB ID: 7C6U) were also performed to compare the conservation of interactions and the ligand’s stability between two different experimentally determined binding modes (Figure S6B). Residues Gly143, Cys145, Glu166 and Gln189 were consistent in their interactions across all replicates, corroborating our noncovalent (Figure 2) and covalent docking data (Figure 4), as well as binding site predictions (Figure S4).

**Figure 6.**
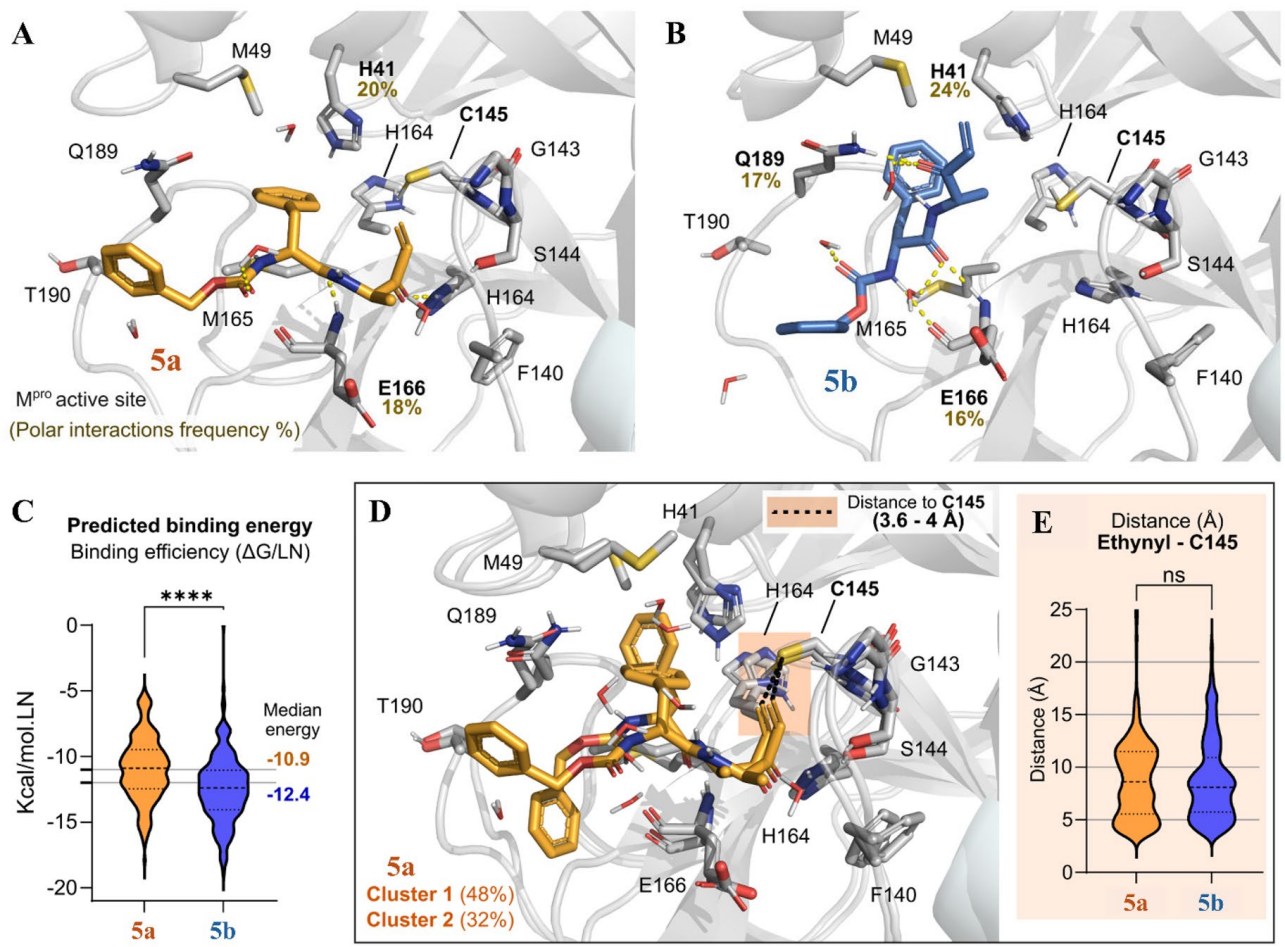
Proposed binding mode of compounds **5a** and **5b** in the active site of SARS-CoV-2 M^pro^ represented by the most populated cluster conformation from the MD simulations for (A) **5a** (bright orange) and (B) **5b** (sky blue). Residues performing polar contacts are labelled in bold and their interaction frequency in the analysed trajectory time (10 x 200 ns per ligand) are shown in olive as percentage values. (C) Violin plot displaying the variation of the calculated binding energy using MM/GBSA along the entire MD trajectory and normalised to the natural log number of heavy atoms (see Methods). Median energy values are highlighted. Differences between distributions were analysed by a one-sample Mann-Whitney multiple comparison test (****p < 0.0001). (D) Most populated clusters depict a short distance (3.6–4 Å) of the reactive vinyl ketone group of **5a** to the catalytic cysteine (orange box). Likewise, **5a** and **5b** are likely to form covalent reactions, even though the average distance of the warhead reactive atoms from the reactive catalytic cysteine sulphur was unstable along the simulations’ trajectory (E).

Subsequently, we performed a combination of multiple short noncovalent MD simulations, that is, 10 replicas of 200 ns, i.e. 2 µs, for each ligand, to identify any binding differences between **5a** and **5b**. These calculations were followed by clustering, geometry and binding energy analysis along the calculated trajectories (Figure 6 and Figure S7). The proposed binding modes of both compounds are represented by the most populated cluster conformation from the MD simulations for **5a** (Figure 6A) and **5b** (Figure 6B). Both configurations rely on hydrogen interactions between the amide linker of the inhibitor and the backbone of Glu166, corroborating our docking results (Figures 2A, 2B, 4A, and 4B), as well as π-mediated interactions between the phenyl ring of the inhibitor and the His41 sidechain. In addition, compound **5b** does not stably occupy the S1 pocket (Cys145 and Gly143) compared to **5a**, but rather interacts with the S3 and S4 pockets, with additional polar contacts with Gln189, as predicted by our docking results (Figures 2B and 4B). These additional interactions are reflected in the predicted lower binding energy values for **5b** compared to **5a** from our MM/GBSA calculations (Figure 6C), consistent with the lower IC_50_ values for **5b** in the enzyme-inhibition assays (Table 1). These simulations also suggest that both compounds are likely to establish a covalent reaction, considering the predicted short distance (3.6 − 4 Å) between the catalytic cysteine and the reactive vinyl ketone group of compound **5a**, and the latter’s position that is prone to nucleophilic attack in the most populated clusters (Figure 6D, orange box). Further, because the distance between the reactive atoms of the vinyl ketone warhead of **5a** and **5b** and the sulphur of the catalytic cysteine was unstable along the simulation trajectory (Figure 6E), the data are consistent with their reversible covalent inhibition in the enzymatic assays (Figures 1 and 3).

Finally, additional methods, such as DFT^104^, should be employed to describe electrophilic warhead effects, especially because DFT is better suited for analysing chemical reactivity as even different warheads can be predicted to have similar outcomes in MD simulations^57^^,^^81^. Thus, we performed DFT calculations using two different basis sets to compare our enantiomers with known reversible and irreversible covalent inhibitors (Table 2). Our results using B3LYP/6–311++G** for **5a** and **5b** (−15.59 to −15.97 kcal/mol) are closer to those for nirmatrelvir (−18.89 kcal/mol) than the irreversible inhibitor, GC373 (−20.14 kcal/mol). A similar behaviour was calculated using B3LYP/6-31G*, with an exception to nirmatrelvir’s values (−8.14 kcal/mol). This behaviour may be due to the differences in the two basis sets, as 6–311++G** is a larger and more complete set than 6–31 G*^105^, which is usually less reliable for comparing electronic effects involved in intermolecular interactions between ligands and molecular targets, and their respective energies^106^. Overall, these results are consistent with the reversible covalent behaviour predicted in our MD simulations for the binding of the vinyl ketone electrophilic group of **5a** and **5b** to the catalytic cysteine residue (Figure 6), as well as the short distance (3.6 − 4 Å) for that covalent reaction (Figures 2A, 2B and 6D). In addition, the lower binding energy calculated from our DFT calculations for **5b** compared to **5a** is consistent with the lower median energy predicted for **5b** in our MM/GBSA (Figure 6C), which has previously been used to evaluate the modulation of electrophilic groups on the covalent inhibition by other SARS-CoV-2 M^pro^ inhibitors^104^. Last, the more thermodynamically favourable behaviour observed for GC373 is consistent with its irreversibility, compared to the reversibility measured for **5a**, **5b** and nirmatrelvir in our rapid dilution assay (Figure 5).

**Table 2.** DFT calculations corroborate the covalent reaction binding energy (kcal/mol) of compounds.

B3LYP	Compound	E_ligand_	E_Cys145_	E_adduct_	E_binding_
6-31G*	**5a**	−792885.67	−453084.25	−1245986.78	**−16.86**
	**5b**	−792885.61	−453084.25	−1245986.97	**−17.11**
	Nirmatrelvir	−1110945.38	−453084.25	−1564037.77	**−8.14**
	GC373	−852466.23	−453084.25	−1305571.91	**−21.43**
6-311++G**	**5a**	−792924.2	−453103.91	−1246043.7	**−15.59**
	**5b**	−792924.44	−453103.91	−1246044.12	**−15.77**
	Nirmatrelvir	−1110996.27	−453103.91	−1564119.07	**−18.89**
	GC373	−852511.12	−453103.91	−1305635.17	**−20.14**

### Compounds 1a, 5a, and 5b are at least two-fold less cytotoxic to L929 than Vero cells

We tested the compounds’ cytotoxicity against two mammalian cells (Table 3). Overall, the three M^pro^ inhibitors were relatively non-toxic to L929 and Vero cells, with CC_50_ values ranging from 74 to 102 µM for L929 cells, and 17 to 49 µM for Vero cells.

**Table 3.** Cytotoxicity of 1a, 5a, and 5b vs. L929 and Vero cells.

	L929	Vero
Inhibitor	CC_50_ (µM)	CC_50_ (µM)
**1a**	73.54 ± 4.34	17.24 ± 0.50
**5a**	101.86 ± 2.06	48.98 ± 3.28
**5b**	85.86 ± 1.69	37.53 ± 2.14
**Ribavirin** ^a^	>100	>100

^a^ Ribavirin data were reported previously for L929^107^ and Vero^72^ cells.

## Discussion

### Discovery of potent SARS-related and MERS-CoV M^pro^ inhibitors in a legacy collection of cysteine protease inhibitors

Given the resistance reported for current SARS-CoV-2 M^pro^ drugs, such as nirmatrelvir and ensitrelvir^30^^,^^32–34^^,^^108^, we aimed to identify new inhibitor starting points from a legacy collection of 141 compounds originally designed to target cruzain, the major cysteine protease of *T. cruzi*. Our screening identified five hits. Two of these, compounds **1a** and **5a**, share a benzyl carbamate moiety, which has been described for cysteine protease inhibitors of SARS-CoV M^pro^^109^, cruzain^47^, hCatL^110^ and a cathepsin L-like protease from the parasitic flatworm, *Fasciola hepatica*^111^. Compounds **1a** and **5a** were also the most potent vs. SARS-CoV-2 M^pro^, but because they had been stored for at least a decade, we resynthesized both, and synthesised the (R,S) version of **5a**, termed **5b**.

Compounds **1a** and **5a** were sub-micromolar inhibitors of the M^pro^ enzymes of SARS-CoV-2, SARS-CoV and MERS-CoV (Table 1). The same was true for **5b** when tested against SARS-CoV and SARS-CoV M^pro^. In fact, of all the hit compounds, **5b** generated the lowest IC_50_ value of 0.073 μM recorded against SARS-CoV M^pro^ (Table 1). Overall, the data are consistent with the greater sequence identity between the two SARS-related M^pro^ enzymes^54^ (96%) compared to MERS-CoV M^pro^ (∼51%; Figure S8). In terms of the structure-activity relationship (SAR), the presence of the ethene-ester group in **1a**, compared to **5a** and **5b** vinyl ketone electrophilic warheads, decreased the IC_50_ value potency between 3- and 9-fold depending on the M^pro^ target (Table 1). Conversely, the presence of the ethene-ester at the P1 position in **1a** resulted in inhibition of hCatL, which was not observed for **5a** or **5b**. The difference in hCatL activity appears to be driven by P1 preferences.It is known that hCatL prefers phenyl residues at the P1 position ^112^. This may contribute to the differing abilities of **2a** (butylbenzene at P1), **3a** (vinylsulfonyl-benzene at P1) and **4a** (methylsulfonyl-benzene at P1) to inhibit hCatL, compared to their weak inhibition of SARS-CoV-2 M^pro^, and their lack of activity against SARS-CoV and MERS-CoV M^pro^.

### Noncovalent and covalent docking, and MD simulations support the reversible and covalent mechanism of 5a and 5b

Given the differences in IC_50_ values for compounds **5a** and **5b** with the M^pro^ targets, we characterised these enzymes’ potential binding sites using FTSite and PrankWeb. Our predictions in relation to the amino acid residues that are engaged by the inhibitors across the different SARS-CoV-2 M^pro^ structures are consistent with those from previous studies^113^^,^^114^. First, FTSite highlighted the MERS-CoV M^pro^ substitutions, Asn142Cys and His164Gln, that would not favour interactions with an inhibitor, as observed for ligands co-crystallized in 270 different structures of SARS-related M^pro^ and MERS-CoV M^pro^^115^. Second, our noncovalent docking using GOLD predicted that the warhead orientations of **5a** and **5b** face away from the catalytic cysteine of MERS-CoV M^pro^, whereas no essential changes were predicted for the two SARS-related proteases. Both predictions support the compounds’ less potent biochemical inhibition of MERS-CoV M^pro^, given this enzyme’s lower overall sequence identity compared to those of the SARS-related coronaviruses. A similar behaviour has been demonstrated for ensitrelvir, which forms hydrogen bonds with Asn142 in the crystal structures of SARS-CoV-2 and SARS-CoV M^pro^ but lacks such bonds with the substituted Cys142 residue in MERS-CoV M^pro^^116^. Third, our covalent docking predictions using Glide XP corroborated the noncovalent docking data and supported the stabilisation of **5a** and **5b** within the pocket, now covalently bound to the catalytic cysteine. These results are consistent with the inhibitors’ covalent inhibition indicated in our biochemical assays.

Our 1 µs MD simulations predicted a lower binding energy (kcal/mol) for compound **5b** compared to **5a**, which agrees with its lower IC_50_ values. Also, the MD simulations suggest that both compounds are likely to establish a covalent reaction, supporting the data from time-dependent and rapid dilution assays. The overall predicted short distance between the nucleophile (Cys145) and the electrophile (ethene) is within the 3–4 Å range often associated with reactive conformations^117^. This proximity and the pose alignment are also consonant with the near-in-line attack proneness typically observed for enzyme catalysis^118^. This propensity for covalent bond formation has also been observed with the MD simulations assessing nirmatrelvir’s reversible binding to the SARS-CoV-2 M^pro^^119^. Finally, to further support the observed behaviour of the two enantiomers, our DFT calculations (Table 2) predicted similar binding formation energies for **5a** and **5b**, equivalent to the MM/GBSA calculations. Values closer to that for the reversible inhibitor, nirmatrelvir, compared to the irreversible inhibitor, GC373, were obtained when using a more complete basis set, 6–311++G**, which is usually more reliable for comparing electronic effects in intermolecular interactions^105^. The data from our MD simulations also suggest that the electrophilic vinyl ketone substituent in **5a** and **5b** forms a reversible covalent bond, as suggested for other inhibitors targeting SARS-CoV-2 M^pro^^4^, such as PBI-0451 (pomotrelvir)^120^ and MI-30^121^, both of which were experimentally validated *in vitro*^122^^,^^123^. Our observation is similar to the reversible bond of the electrophilic nitrile of nirmatrelvir with the catalytic Cys145, a reversible behaviour predicted by MD simulations^119^ and experimentally validated *in vitro*^124^. In further support of the formation of a reversible covalent bond, the covalent modification of a single cysteine residue in the homologous *Toxoplasma gondii* DJ-1 protein has been shown for **5a**^125^.

It has been shown that conserved and stable interactions between inhibitor substituents and residues of SARS-CoV-2 M^pro^, other than the catalytic cysteine, usually correlate with biochemical inhibition and antiviral data^124^. This is also the case for our pose predictions from both the noncovalent and covalent docking data, and MD simulations for the two enantiomers, **5a** and **5b**; specifically, for **5b**, the predicted interactions between the ligand’s P4 benzyl-ester and Gln189, the P3 amide linker and the backbone of Glu166, and the P1 ester with Ser144. Also, previous MD simulations have demonstrated that inhibitor interactions with Gln189, Glu166, Gly143 and His41 of SARS-CoV-2 M^pro^ are postulated to significantly improve inhibition^81^. We observed this for compound **5b** compared to **5a** in our MD simulations, which is also consistent with the differences in their biochemical inhibition. In contrast, neither **5a** or **5b** inhibited hCatL, findings that are supported by our noncovalent docking data, which indicate that the P1 residue faces away from the catalytic Cys25.

The submicromolar inhibition values measured for **5a** and/or **5b** with the three M^pro^ enzymes are just 2–10-fold greater that those determined for nirmatrelvir, which encourages their further chemical modification to improve potency. Future studies might also include the testing of SARS-related virus strains^126^, evaluating the effects of inhibitor-induced target mutations^30^^,^^124^ and the assessment of antiviral activity in Vero cells^127^, considering that the inhibitors are relatively non-cytotoxic (Table 3). Compound **1a**, although less potent against the M^pro^ enzymes, has the advantage of inhibiting hCatL, which is important for viral entry into human cells^43^, and, consequentially, could also be considered a starting point in the design of dual-target inhibitors^128^.

## Conclusions

The design, discovery and development of antiviral drugs that inhibit the SARS-CoV-2 M^pro^ is a continuous endeavour given the emergence of resistance mutants. From a collection of 141 cysteine protease inhibitors that were originally designed to target cruzain, the major cysteine protease of *T. cruzi*, we screened SARS-CoV-2 M^pro^ and PL^pro^ for inhibitor starting points. We identified five (sub)micromolar inhibitors of M^pro^ and none against PL^pro^. These inhibitors also variously inhibited the M^pro^ enzymes of SARS-CoV and MERS-CoV M^pro^, and hCatL, the latter of which is important for coronavirus entry into host cells. The most potent compounds, **1a** and **5a**, and the latter’s (R,S) enantiomer, **5b**, were indicated as covalent and reversible inhibitors of SARS-CoV-2 M^pro^. The biochemical data generated for the inhibitors’ specificity of binding, mechanism of action, and relative inhibition potency, were supported by docking and MD simulations, as well as DFT calculations. This study, combining experimental validation with computational simulations, highlights the utility of revisiting legacy chemical assets to identify promising starting points for the development of new antiviral drug candidates.

## Supplementary Material

CLEAN_rev_anonymous_supplemental_mpro_jeimc_09252025.docx

## Data Availability

Energy measurements, trajectories and interaction data are available in separate reports in the Zenodo repository (10.5281/zenodo.7841336). Crystallographic structures used are available from the Protein Data Bank (PDB) (https://www.rcsb.org/). All third-party software used were as follows: GraphPad Prism version 9.0 (https://www.graphpad.com/) is distributed under licence. Schrödinger Suite 2021.2 (https://www.schrodinger.com) is distributed under licence. PyMOL version 2.5.7 (https://pymol.org/) is distributed under licence. Maestro version v2023.1 (https://newsite.schrodinger.com/platform/products/maestro/) is distributed under the Schrödinger’s licence. Discovery Studio (https://www.3ds.com/products/biovia/discovery-studio) is distributed under licence. OMEGA version 3.1.1.2 (https://www.eyesopen.com/omega) and QUACPAC version 2.0.1.2 (https://www.eyesopen.com/quacpac) are distributed under the OpenEye Scientific licence.

## CRediT roles

Conceptualisation, C.R.C. and A.J.O.; data curation, M.S.M.S., T.K., K.R.F., E.V.S.R., E.G.O., F.K.M.O., I.S.F., T.H.M.F., E.B.S., P.F., D.E.S., R.O.S., J.L.S.N., A.P., B.E.F.M., J.G.A.C.R., J.S.A., V.G.M., A.J.O. and C.R.C.; formal analysis, M.S.M.S., T.K., K.R.F., E.V.S.R., E.G.O., J.L.S.N., A.P., B.E.F.M., J.G.A.C.R., J.S.A., V.G.M., A.J.O. and C.R.C.; funding acquisition, M.S.M.S., T.K., J.L.S.N., A.P., B.E.F.M., J.G.A.C.R., J.S.A., V.G.M., A.J.O., and C.R.C.; investigation, M.S.M.S., T.K., K.R.F., E.V.S.R., E.G.O., F.K.M.O., I.S.F., T.H.M.F., E.B.S., P.F., D.E.S., R.O.S., and V.G.M.; methodology, M.S.M.S., T.K., K.R.F., E.V.S.R., E.G.O., F.K.M.O., E.B.S., P.F., D.E.S., R.O.S., J.L.S.N., A.P., B.E.F.M., J.G.A.C.R., V.G.M., A.J.O. and C.R.C.; project administration, M.S.M.S., B.E.F.M., J.G.A.C.R., J.S.A., V.G.M., A.J.O. and C.R.C.; software, M.S.M.S., T.K., A.P. and V.G.M.; resources, M.S.M.S., T.K., D.E.S., R.S., J.L.S.N., A.P., B.E.F.M., J.G.A.C.R., J.S.A., V.G.M., A.J.O. and C.R.C.; supervision, T.K., B.E.F.M., J.G.A.C.R., J.S.A., V.G.M., A.J.O. and C.R.C.; validation, M.S.M.S., T.K., K.R.F., E.B.S., P.F., J.G.A.C.R.,V.G.M., A.J.O. and C.R.C.; visualisation, M.S.M.S., T.K., K.R.F., E.V.S.R., E.G.O., F.K.M.O., I.S.F., T.H.M.F., B.E.F.M., J.G.A.C.R., V.G.M., A.J.O. and C.R.C.; writing – original draft, M.S.M.S., T.K., K.R.F., E.G.O., B.E.F.M., J.G.A.C.R., J.S.A., V.G.M., A.J.O. and C.R.C.; writing – review & editing, M.S.M.S., T.K., K.R.F., E.V.S.R., E.G.O., F.K.M.O., I.S.F., T.H.M.F., E.B.S., P.F., D.E.S., R.O.S., J.L.S.N., A.P., B.E.F.M., J.G.A.C.R., J.S.A., V.G.M., A.J.O. and C.R.C. All authors have read and agreed to the final version of the manuscript.
