# Supplementary material for: Discovery of benzyl carbamate inhibitors of coronavirus Mpro enzymes from a legacy collection of cysteine protease inhibitors
Source: J Enzyme Inhib Med Chem. 2025 Nov 17;40(1):2585619. doi: 10.1080/14756366.2025.2585619 (PMC12624906; doi:10.1080/14756366.2025.2585619)
Supplement: CLEAN_rev_anonymous_supplemental_mpro_jeimc_09252025.docx [file IENZ_A_2585619_SM8541.docx]

**Supplemental Material**

**Discovery of benzyl carbamate inhibitors of coronavirus M^pro^ enzymes from a legacy collection of cysteine protease inhibitors**

The supplemental material comprises the following information:

Table S1. Percentage inhibition of SARS-CoV-2 M^pro^ activity by 141 compounds designed to cruzain.

Table S2. Percentage inhibition of SARS-CoV-2 PL^pro^ activity by 141 compounds designed to cruzain.

Table S3. SARS-CoV-2, SARS-CoV and MERS-CoV M^pro^ residues predicted with PrankWeb to be favorable for ligand interactions.

Figure S1. Michaelis-Menten plot with the substrate Ac-Abu-Tle-Leu-Gln-MCA (Biosynth, FA178674) for SARS-CoV-2 M^pro^.

Figure S2. Concentration-response curves of hits against SARS-CoV-2 M^pro^ and hCatL.

Figure S3. Concentration-response curves of (re)synthesized compounds against SARS-CoV and MERS-CoV M^pro^.

Figure S4. Predicted binding site residues in different M^pro^ enzymes.

Figure S5. Redocking of M^pro^ and hCatL co-crystallized ligands using GOLD.

Figure S6. Noncovalent MD simulations of the co-crystallized ligand GC376 in SARS-CoV-2 M^pro^.

Figure S7. RMSD and RMSF values for **5a** (A and B, respectively) and **5b** (C and D, respectively).

Figure S8. Alignment of different coronavirus M^pro^ sequences (SARS-CoV-2, SARS-CoV, and MERS-CoV).

Chemistry for the synthesis of compounds **ii**, **iii**, **iv**, **1a**, **vi**, **vii**, **5a**, and **5b** (Schemes 1 and 2) and single crystal structure reports for compounds **5a** and **5b** (Tables A – E).

**Table S1. Percentage inhibition of SARS-CoV-2 M^pro^ activity by 141 compounds designed to cruzain**

| **Compound** | **% inhibition**  **(10 μM^a^)** | **Compound** | **% inhibition**  **(10 μM^a^)** | **Compound** | **% inhibition**  **(10 μM^a^)** |
| --- | --- | --- | --- | --- | --- |
| 1 / WRR-129 | 0.0 ± 0.0 | 49 / WRR-281 | 0.00 ± 0.00 | 97 / WRR-382 | 13.15 ± 2.5 |
| 2 / WRR-130 | 0.0 ± 0.0 | 50 / WRR-286 | 0.00 ± 0.00 | 98 / WRR-384 | 19.24 ± 4.18 |
| 3 / WRR-139 | 0.0 ± 0.0 | 51 / WRR-287 | 0.34 ± 0.34 | ***99 / WRR-385 / 3a*** | ***51.55 ± 1.38*** |
| 4 / WRR-145 | 0.0 ± 0.0 | 52 / WRR-288 | 1.41 ± 0.73 | 100 / WRR-389 | 3.65 ± 3.65 |
| 5 / WRR-146 | 0.0 ± 0.0 | 53 / WRR-292 | 0.00 ± 0.00 | 101 / WRR-390 | 11.07 ± 5.85 |
| 6 / WRR-148 | 0.8 ± 0.8 | 54 / WRR-293 | 1.62 ± 1.15 | 102 / WRR-391 | 7.11 ± 3.63 |
| 7 / WRR-150 | 0.0 ± 0.0 | 55 / WRR-297 | 1.19 ± 0.83 | 103 / WRR-392 | 10.04 ± 3.27 |
| 8 / WRR-152 | 2.98 ± 0.29 | 56 / WRR-299 | 2.29 ± 1.25 | 104 / WRR-397 | 6.08 ± 3.1 |
| 9 / WRR-153 | 0.08 ± 0.08 | 57 / WRR-300 | 5.96 ± 2.9 | 105 / WRR-398 | 5.26 ± 2.72 |
| 10 / WRR-157 | 0.0 ± 0.0 | 58 / WRR-301 | 1.7 ± 0.96 | 106 / WRR-400 | 3.30 ± 2.11 |
| 11 / WRR-158 | 0.0 ± 0.0 | 59 / WRR-302 | 11.02 ± 2.36 | 107 / WRR-412 | 1.07 ± 0.68 |
| 12 / WRR-159 | 0.08 ± 0.08 | 60 / WRR-303 | 0.34 ± 0.34 | 108 / WRR-413 | 0.00 ± 0.00 |
| 13 / WRR-160 | 0.92 ± 0.92 | 61 / WRR-304 | 1.02 ± 0.59 | 109 / WRR-414 | 0.37 ± 0.37 |
| 14 / WRR-161 | 0.0 ± 0.0 | 62 / WRR-305 | 2.54 ± 1.46 | 110 / WRR-415 | 1.3 ± 1.3 |
| 15 / WRR-163 | 0.00 ± 0.00 | 63 / WRR-306 | 0.59 ± 0.59 | 111 / WRR-416 | 1.58 ± 0.88 |
| 16 / WRR-164 | 0.25 ± 0.25 | 64 / WRR-307 | 0.00 ± 0.00 | 112 / WRR-417 | 0.00 ± 0.00 |
| 17 / WRR-166 | 1.27 ± 0.8 | 65 / WRR-308 | 2.17 ± 1.44 | 113 / WRR-418 | 2.97 ± 1.68 |
| 18 / WRR-168 | 4.05 ± 1.27 | 66 / WRR-309 | 0.76 ± 0.52 | 114 / WRR-419 | 0.00 ± 0.00 |
| 19 / WRR-169 | 12.53 ± 0.26 | 67 / WRR-310 | 2.72 ± 1.38 | 115 / WRR-431 | 0.00 ± 0.00 |
| 20 / WRR-170 | 0.00 ± 0.00 | 68 / WRR-311 | 3.97 ± 1.94 | 116 / WRR-436 | 1.57 ± 1.57 |
| 21 / WRR-171 | 0.00 ± 0.00 | 69 / WRR-312 | 2.38 ± 2.38 | ***117 / WRR-438 / 4a*** | ***52.55 ± 3.61*** |
| 22 / WRR-172 | 0.00 ± 0.00 | 70 / WRR-315 | 4.82 ± 4.58 | 118 / WRR-439 | 3.99 ± 2.61 |
| 23 / WRR-173 | 0.36 ± 0.36 | 71 / WRR-316 | 4.94 ± 2.71 | 119 / WRR-441 | 5.75 ± 2.72 |
| 24 / WRR-174 | 1.59 ± 1.59 | ***72 / WRR-317 / 2a*** | ***53.97 ± 1.34*** | 120 / WRR-442 | 7.55 ± 4.94 |
| 25 / WRR-176 | 0.00 ± 0.00 | 73 / WRR-318 | 30.16 ± 0.88 | 121 / WRR-443 | 2.87 ± 1.4 |
| 26 / WRR-177 | 7.45 ± 0.6 | 74 / WRR-319 | 4.07 ± 4.07 | 122 / WRR-444 | 1.94 ± 1.94 |
| 27 / WRR-179 | 2.17 ± 2.17 | 75 / WRR-320 | 4.71 ± 2.54 | 123 / WRR-445 | 5.07 ± 2.54 |
| ***28 / WRR-180 / 1a*** | ***96.75 ± 1.04*** | 76 / WRR-333 | 1.0 ± 0.87 | 124 / WRR-446 | 0.00 ± 0.00 |
| ***29 / WRR-181 / 5a*** | ***100.00 ± 0.00*** | 77 / WRR-342 | 6.93 ± 3.5 | 125 / WRR-447 | 5.26 ± 2.64 |
| 30 / WRR-182 | 0.00 ± 0.00 | 78 / WRR-343 | 0.67 ± 0.67 | 126 / WRR-453 | 0.00 ± 0.00 |
| 31 / WRR-183 | 0.00 ± 0.00 | 79 / WRR-346 | 5.37 ± 2.77 | 127 / WRR-454 | 2.77 ± 1.38 |
| 32 / WRR-193 | 0.81 ± 0.81 | 80 / WRR-347 | 9.33 ± 5.5 | 128 / WRR-459 | 0.00 ± 0.00 |
| 33 / WRR-195 | 0.00 ± 0.00 | 81 / WRR-350 | 0.83 ± 0.83 | 129 / WRR-460 | 0.00 ± 0.00 |
| 34 / WRR-196 | 0.54 ± 0.54 | 82 / WRR-352 | 3.59 ± 1.98 | 130 / WRR-463 | 2.61 ± 2.61 |
| 35 / WRR-197 | 0.00 ± 0.00 | 83 / WRR-353 | 3.29 ± 1.85 | 131 / WRR-464 | 1.23 ± 1.23 |
| 36 / WRR-198 | 0.00 ± 0.00 | 84 / WRR-356 | 1.74 ± 1.09 | 132 / WRR-465 | 0.84 ± 0.84 |
| 37 / WRR-200 | 0.45 ± 0.45 | 85 / WRR-358 | 1.39 ± 1.39 | 133 / WRR-466 | 2.53 ± 2.53 |
| 38 / WRR-201 | 0.00 ± 0.00 | 86 / WRR-360 | 4.23 ± 2.68 | 134 / WRR-467 | 3.87 ± 2.44 |
| 39 / WRR-202 | 0.09 ± 0.09 | 87 / WRR-361 | 2.69 ± 2.05 | 135 / WRR-468 | 2.95 ± 2.25 |
| 40 / WRR-203 | 0.00 ± 0.00 | 88 / WRR-366 | 2.32 ± 2.32 | 136 / WRR-477 | 0.23 ± 0.23 |
| 41 / WRR-209 | 0.00 ± 0.00 | 89 / WRR-368 | 1.19 ± 0.72 | 137 / WRR-478 | 0.00 ± 0.00 |
| 42 / WRR-229 | 0.00 ± 0.00 | 90 / WRR-370 | 6.02 ± 3.56 | 138 / WRR-479 | 0.23 ± 0.23 |
| 43 / WRR-230 | 0.36 ± 0.36 | 91 / WRR-372 | 4.70 ± 2.39 | 139 / WRR-482 | 2.01 ± 1.66 |
| 44 / WRR-235 | 0.97 ± 0.97 | 92 / WRR-376 | 4.33 ± 2.51 | 140 / WRR-500 | 1.15 ± 1.15 |
| 45 / WRR-236 | 0.43 ± 0.43 | 93 / WRR-377 | 4.37 ± 2.22 | 141 / WRR-605 | 5.65 ± 1.18 |
| 46 / WRR-241 | 0.00 ± 0.00 | 94 / WRR-378 | 8.82 ± 4.17 | Nirmatrelvir* | 95.78 ± 2.12 |
| 47 / WRR-261 | 0.00 ± 0.00 | 95 / WRR-379 | 6.55 ± 3.96 | - | - |
| 48 / WRR-267 | 0.00 ± 0.00 | 96 / WRR-381 | 11.42 ± 3.67 | - | - |

^a^ Percentage inhibition is reported as the mean and standard error calculated from one assay performed in triplicate. Errors are given by the ratio of the standard deviation to the square root of the number of measurements (*n* = 3). Initial hits are highlighted in bold and italics. WRR: William R. Roush. *The M^pro^ inhibitor, nirmatrelvir, used as a positive control, was tested at 100 nM.

**Table S2. Percentage inhibition of SARS-CoV-2 PL^pro^ activity by 141 compounds designed to cruzain**

| **Compound** | **% inhibition**  **(10 μM^a^)** | **Compound** | **% inhibition**  **(10 μM^a^)** | **Compound** | **% inhibition**  **(10 μM^a^)** |
| --- | --- | --- | --- | --- | --- |
| 1 / WRR-129 | 0.0 ± 0.0 | 49 / WRR-281 | 0.0 ± 0.0 | 97 / WRR-382 | 2.7 ± 1.57 |
| 2 / WRR-130 | 5.67 ± 9.06 | 50 / WRR-286 | 0.0 ± 0.0 | 98 / WRR-384 | 4.39 ± 3.51 |
| 3 / WRR-139 | 0.0 ± 0.0 | 51 / WRR-287 | 0.0 ± 0.0 | 99 / WRR-385 / 3a | 1.84 ± 2.85 |
| 4 / WRR-145 | 7.55 ± 5.65 | 52 / WRR-288 | 0.0 ± 0.0 | 100 / WRR-389 | 3.37 ± 9.18 |
| 5a / WRR-146 | 0.0 ± 0.0 | 53 / WRR-292 | 0.0 ± 0.0 | 101 / WRR-390 | 0.78 ± 4.65 |
| 6a / WRR-148 | 11.77 ± 6.54 | 54 / WRR-293 | 0.0 ± 0.0 | 102 / WRR-391 | 1.25 ± 4.18 |
| 7a / WRR-150 | 2.33 ± 0.82 | 55 / WRR-297 | 2.38 ± 7.03 | 103 / WRR-392 | 3.16 ± 7.02 |
| 8a / WRR-152 | 9.01 ± 7.25 | 56 / WRR-299 | 7.3 ± 2.49 | 104 / WRR-397 | 0.0 ± 0.0 |
| 9a / WRR-153 | 0.0 ± 0.0 | 57 / WRR-300 | 0.0 ± 0.0 | 105 / WRR-398 | 0.0 ± 0.0 |
| 10a / WRR-157 | 9.37 ± 7.47 | 58 / WRR-301 | 0.0 ± 0.0 | 106 / WRR-400 | 0.0 ± 0.0 |
| 11 / WRR-158 | 0.0 ± 0.0 | 59 / WRR-302 | 0.0 ± 0.0 | 107 / WRR-412 | 0.0 ± 0.0 |
| 12 / WRR-159 | 3.99 ± 7.07 | 60 / WRR-303 | 0.0 ± 0.0 | 108 / WRR-413 | 0.0 ± 0.0 |
| 13 / WRR-160 | 21.06 ± 2.32 | 61 / WRR-304 | 0.0 ± 0.0 | 109 / WRR-414 | 0.0 ± 0.0 |
| 14 / WRR-161 | 4.96 ± 6.61 | 62 / WRR-305 | 0.0 ± 0.0 | 110 / WRR-415 | 0.0 ± 0.0 |
| 15 / WRR-163 | 0.0 ± 0.0 | 63 / WRR-306 | 0.0 ± 0.0 | 111 / WRR-416 | 0.0 ± 0.0 |
| 16 / WRR-164 | 9.15 ± 9.18 | 64 / WRR-307 | 4.54 ± 2.33 | 112 / WRR-417 | 0.0 ± 0.0 |
| 17 / WRR-166 | 0.0 ± 0.0 | 65 / WRR-308 | 7.31 ± 4.81 | 113 / WRR-418 | 2.69 ± 6 |
| 18 / WRR-168 | 0.0 ± 0.0 | 66 / WRR-309 | 4.14 ± 2.26 | 114 / WRR-419 | 0.0 ± 0.0 |
| 19 / WRR-169 | 0.0 ± 0.0 | 67 / WRR-310 | 0.0 ± 0.0 | 115 / WRR-431 | 1.63 ± 2.19 |
| 20 / WRR-170 | 3.56 ± 7.79 | 68 / WRR-311 | 0.84 ± 0.84 | 116 / WRR-436 | 0.05 ± 2.89 |
| 21 / WRR-171 | 2.33 ± 3.3 | 69 / WRR-312 | 7.61 ± 2.77 | 117 / WRR-438 / 4a | 1.56 ± 4.68 |
| 22 / WRR-172 | 7.58 ± 8.22 | 70 / WRR-315 | 38.07 ± 1.49 | 118 / WRR-439 | 0.0 ± 0.0 |
| 23 / WRR-173 | 0.46 ± 2.41 | 71 / WRR-316 | 10.14 ± 6.2 | 119 / WRR-441 | 0.0 ± 0.0 |
| 24 / WRR-174 | 0.0 ± 0.0 | 72 / WRR-317 / 2a | 5.2 ± 1.23 | 120 / WRR-442 | 0.0 ± 0.0 |
| 25 / WRR-176 | 3.43 ± 1.91 | 73 / WRR-318 | 7.23 ± 3.98 | 121 / WRR-443 | 5.71 ± 0.73 |
| 26 / WRR-177 | 0.0 ± 0.0 | 74 / WRR-319 | 1.1 ± 0.59 | 122 / WRR-444 | 0.0 ± 0.0 |
| 27 / WRR-179 | 3.51 ± 1.8 | 75 / WRR-320 | 7.56 ± 3.14 | 123 / WRR-445 | 0.0 ± 0.0 |
| 28 / WRR-180 / 1a | 0.0 ± 0.0 | 76 / WRR-333 | 7.34 ± 1.91 | 124 / WRR-446 | 0.0 ± 0.0 |
| 29 / WRR-181 / 5a | 6.14 ± 2.42 | 77 / WRR-342 | 3.92 ± 3.19 | 125 / WRR-447 | 0.57 ± 0.47 |
| 30 / WRR-182 | 10.92 ± 5.5 | 78 / WRR-343 | 7.54 ± 3.39 | 126 / WRR-453 | 0.0 ± 0.0 |
| 31 / WRR-183 | 3.86 ± 4.46 | 79 / WRR-346 | 5.35 ± 3.08 | 127 / WRR-454 | 0.0 ± 0.0 |
| 32 / WRR-193 | 0.0 ± 0.0 | 80 / WRR-347 | 5.08 ± 1.37 | 128 / WRR-459 | 4.42 ± 3.61 |
| 33 / WRR-195 | 1.82 ± 5.8 | 81 / WRR-350 | 2.58 ± 2.58 | 129 / WRR-460 | 0.0 ± 0.0 |
| 34 / WRR-196 | 4.53 ± 7.37 | 82 / WRR-352 | 1.84 ± 0.97 | 130 / WRR-463 | 0.0 ± 0.0 |
| 35 / WRR-197 | 3.89 ± 2.2 | 83 / WRR-353 | 2.8 ± 3.88 | 131 / WRR-464 | 0.0 ± 0.0 |
| 36 / WRR-198 | 7.5 ± 8.23 | 84 / WRR-356 | 0.0 ± 0.0 | 132 / WRR-465 | 0.0 ± 0.0 |
| 37 / WRR-200 | 3.45 ± 2.73 | 85 / WRR-358 | 0.0 ± 0.0 | 133 / WRR-466 | 0.0 ± 0.0 |
| 38 / WRR-201 | 0.0 ± 0.0 | 86 / WRR-360 | 2.09 ± 3.12 | 134 / WRR-467 | 0.0 ± 0.0 |
| 39 / WRR-202 | 0.0 ± 0.0 | 87 / WRR-361 | 0.0 ± 0.0 | 135 / WRR-468 | 1.89 ± 1.77 |
| 40 / WRR-203 | 0.67 ± 1.85 | 88 / WRR-366 | 0.0 ± 0.0 | 136 / WRR-477 | 0.0 ± 0.0 |
| 41 / WRR-209 | 0.18 ± 0.75 | 89 / WRR-368 | 0.0 ± 0.0 | 137 / WRR-478 | 13.58 ± 2.86 |
| 42 / WRR-229 | 0.0 ± 0.0 | 90 / WRR-370 | 2.32 ± 6.02 | 138 / WRR-479 | 0.0 ± 0.0 |
| 43 / WRR-230 | 0.0 ± 0.0 | 91 / WRR-372 | 0.0 ± 0.0 | 139 / WRR-482 | 0.0 ± 0.0 |
| 44 / WRR-235 | 0.0 ± 0.0 | 92 / WRR-376 | 0.0 ± 0.0 | 140 / WRR-500 | 2.61 ± 4.37 |
| 45 / WRR-236 | 0.0 ± 0.0 | 93 / WRR-377 | 0.0 ± 0.0 | 141 / WRR-605 | 0.0 ± 0.0 |
| 46 / WRR-241 | 0.0 ± 0.0 | 94 / WRR-378 | 4.18 ± 3.99 | GRL-0617 | 90.3 ± 1.03 |
| 47 / WRR-261 | 0.0 ± 0.0 | 95 / WRR-379 | 1.3 ± 4.68 | - | - |
| 48 / WRR-267 | 0.0 ± 0.0 | 96 / WRR-381 | 1.45 ± 3.06 | - | - |

^a^ Percentage inhibition is reported as the mean and standard error calculated from one assay performed in triplicate. Errors are given by the ratio of the standard deviation to the square root of the number of measurements (*n* = 3). WRR: William R. Roush.

**Table S3. SARS-CoV-2, SARS-CoV and MERS-CoV M^pro^ residues predicted with PrankWeb to be favorable for ligand interactions**

| **Target** | **Site #1**  **Score (probability, %)** | **Site #2**  **Score (probability, %)** | **Site #3**  **Score (probability, %)** |
| --- | --- | --- | --- |
| **SARS-CoV-2**  **(PDB 7D1M)** | 15.78 (60.5%) | 14.71 (56.9%) | 11.67 (44.5%) |
| **Residues** | B: Thr25, Thr26, Leu27, His41, Met49, Phe140, Leu141, Asn142, Gly143, Ser144, Cys145, His163, His164, Met165, Glu166, Asp187, Arg188, Gln189 | A: Thr25, Thr26, Leu27, His41, Met49, Phe140, Asn142, Gly143, Ser144, Cys145, His163, His164, Met165, Glu166, Asp187, Arg188, Gln189 | A: Gly2, Phe3, Arg4, Asn214, Gln299, Gly302, Val303, Thr304, Gln306  B: Lys137, Gly138, Ser139, Phe140, Leu141, Glu166, Thr169, Gly170, Val171, His172 |
| **SARS-CoV**  **(PDB 6W2A)** | 13.19 (51.0%) | 12.65 (48.9%) | 12.11 (46.6%) |
| **Residues** | A: Ala116, Tyr118, Ser123, Gly124, Ser139, Leu141  B: Met6, Ala7, Phe8, Thr111, Gln127, Asn151, Ile152, Thr292, Phe294, Asp295, Arg298, Gln299 | A: Thr25, Thr26, Leu27, His41, Met49, Phe140, Leu141, Asn142, Gly143, Ser144, Cys145, His163, His164, Met165, Glu166, His172, Asp187, Arg188, Gln189 | B: Thr25, Thr26, Leu27, His41, Met49, Phe140, Leu141, Asn142, Gly143, Ser144, Cys145, His163, His164, Met165, Glu166, Asp187, Arg188, Gln189 |
| **MERS-CoV**  **(PDB 6VH3)** | 13.20 (51.1%) | 10.49 (38.9%) | 9.44 (33.8%) |
| **Residues** | B: Ser24, Met25, Thr26, His41, Leu49, Phe140, Leu141, Cys142, Gly143, Ser144, Cys145, His163, Gln164, Met165, Glu166, Asp187, Lys188, Gln189 | A: Ala116, Tyr118, Thr123, Ser129, Leu141  B: Met6, Ser7, His8, Ala111, Phe112, Ser113, Thr127, Thr151, Lys152, Glu153, Thr289, Glu291, Asp292, Met295, Gln296, Gly299 | A: Met25, Thr26, His41, Leu49, Phe140, Leu141, Cys142, Gly143, Ser144, Cys145, His163, Gln164, Met165, Glu166, His172, Asp187, Lys188, Gln189 |

A: monomer A; B: monomer B.


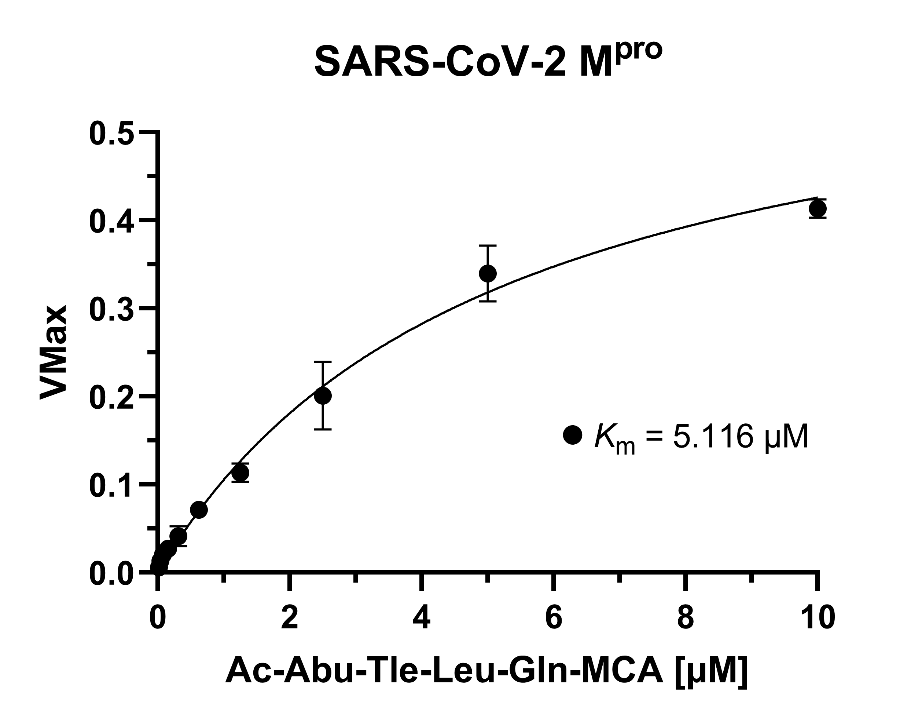


**Figure S1**. Michaelis-Menten plot with the substrate, Ac-Abu-Tle-Leu-Gln-MCA (Biosynth, FA178674) for SARS-CoV-2 M^pro^. The catalysis was monitored continuously for 2 h at 37 °C using a Synergy HTX (BioTek, Winooski, VT, USA) microplate reader with excitation/emission wavelengths of 360/460 nm. Ten different compound concentrations (0.0195 - 10 µM) were assessed in one assay that was performed in triplicate (*n* = 3 data points). V_max_ values were considered for calculation of the substrate *K*_m_.





**Figure S2**. Concentration-response curves of hits against SARS-CoV-2 M^pro^ and hCatL. At least eight different compound concentrations were assessed for SARS-CoV-2 M^pro^ (0.000976 – 40 µM) and hCatL (0.0000195 – 80 μM). The IC_50_ values (means ± standard errors) were calculated by nonlinear regression (r^2^ > 0.9) of data from two independent assays, each performed in triplicate (*n* = 6 data points). Compounds **1a**, **5a** and **5b** were (re)synthesized for these assays.


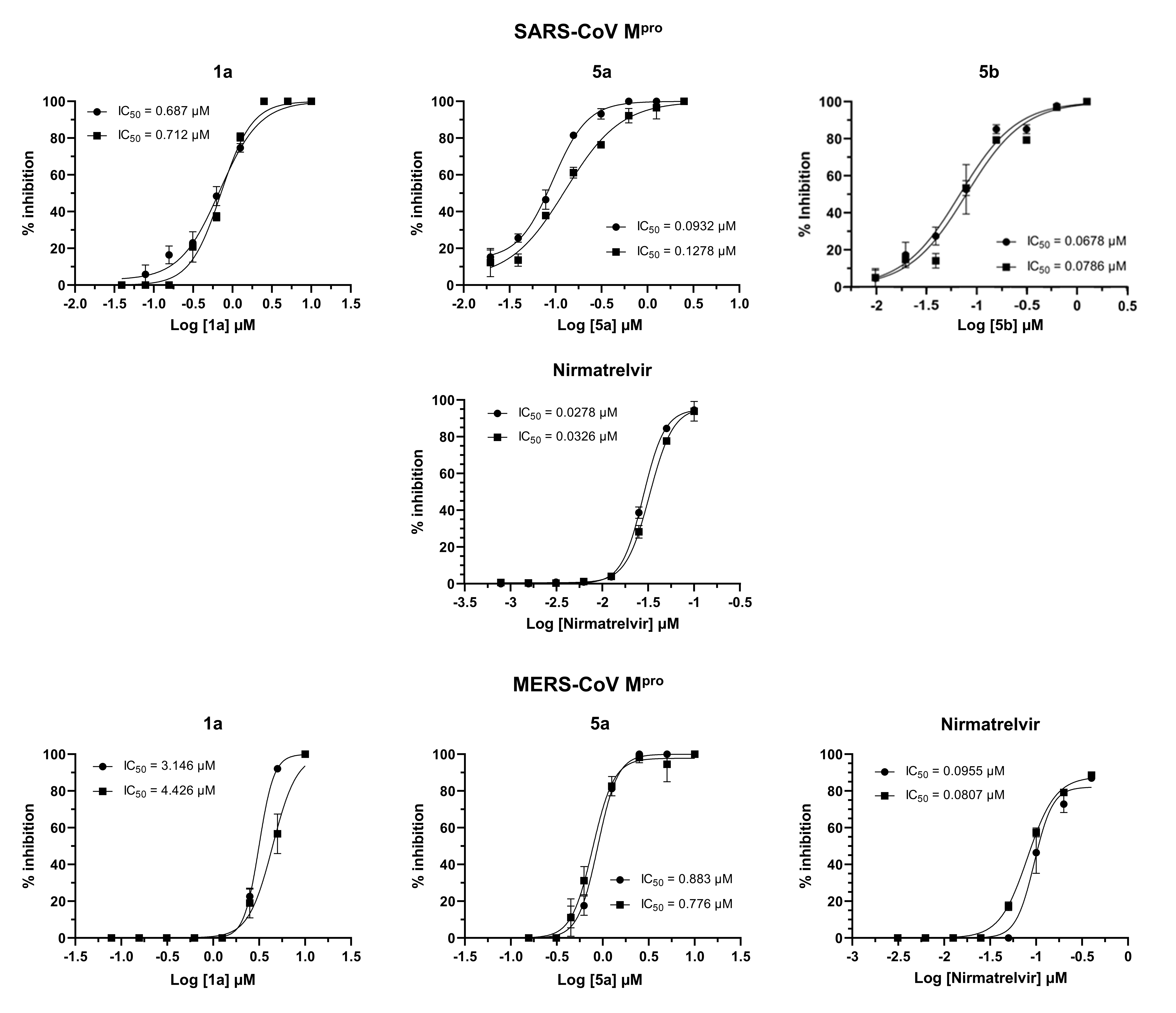


**Figure S3.** Concentration-response curves of (re)synthesized compounds against SARS-CoV and MERS-CoV M^pro^ enzymes. At least eight different compound concentrations (0.00078 - 10 µM) were assessed. The IC_50_ values (means ± standard errors) were calculated by nonlinear regression (r^2^ > 0.9) of data from two independent assays, each performed in triplicate (*n* = 6 data points).


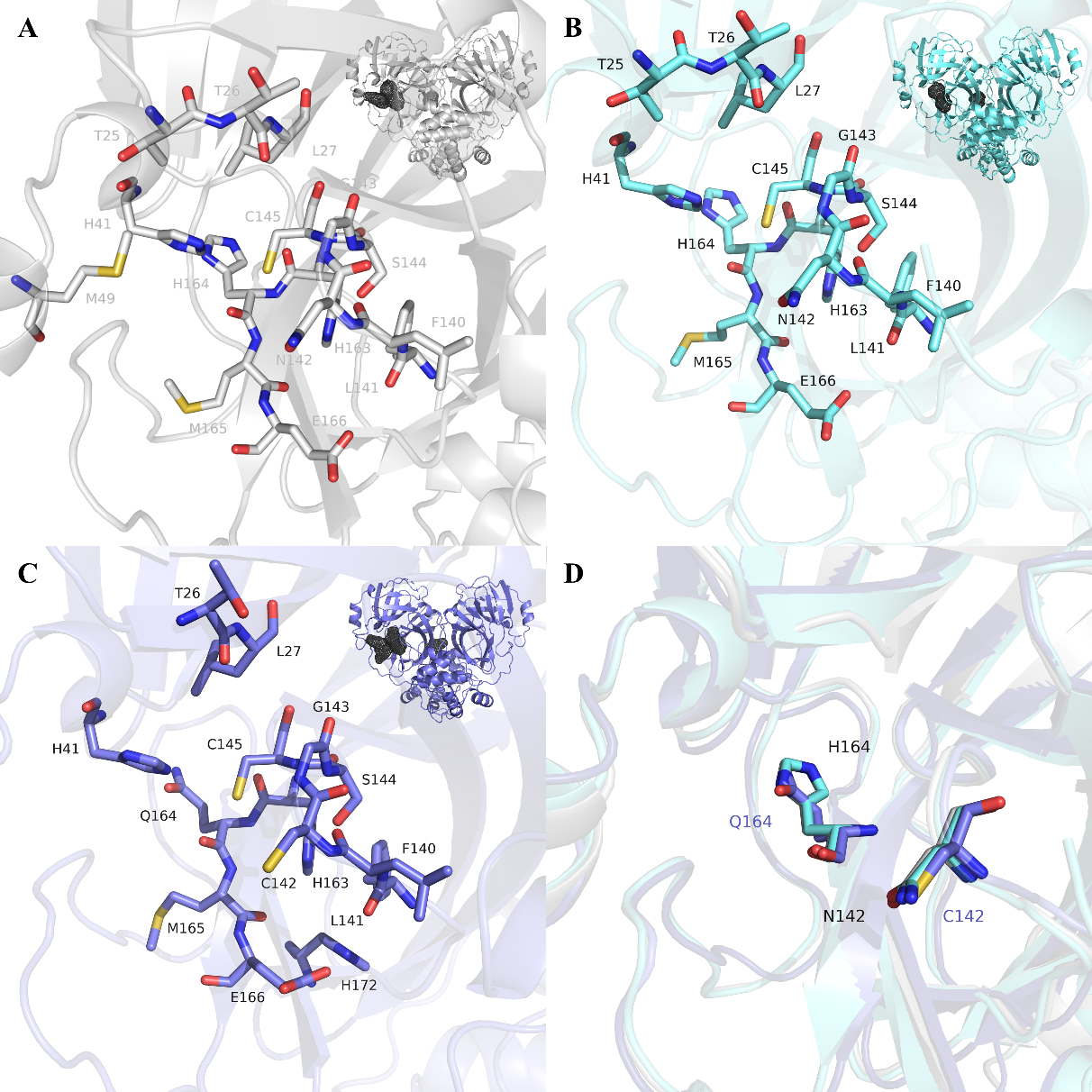


**Figure S4.** Predicted binding site residues in different M^pro^ enzymes. Predictions using FTSite resulted in potential residues for interaction with ligands displayed as 3D black meshes (image inserts, top right) in (A) SARS-CoV-2 (white), (B) SARS-CoV (aquamarine) and (C) MERS-CoV (slate blue). The catalytic site residues, His41 and Cys145, as well as Thr26, Leu27, Phe140, Leu141, Asn142/Cys142, Gly143, Ser144, His163, His164/Gln164, Met165 and Glu166 were predicted as potential binding residues. (D) Superimposed structures display different conformations due to the substitutions, Asn142/Cys142 and His164/Gln164. Images were generated with PyMOL (v2.5.7).


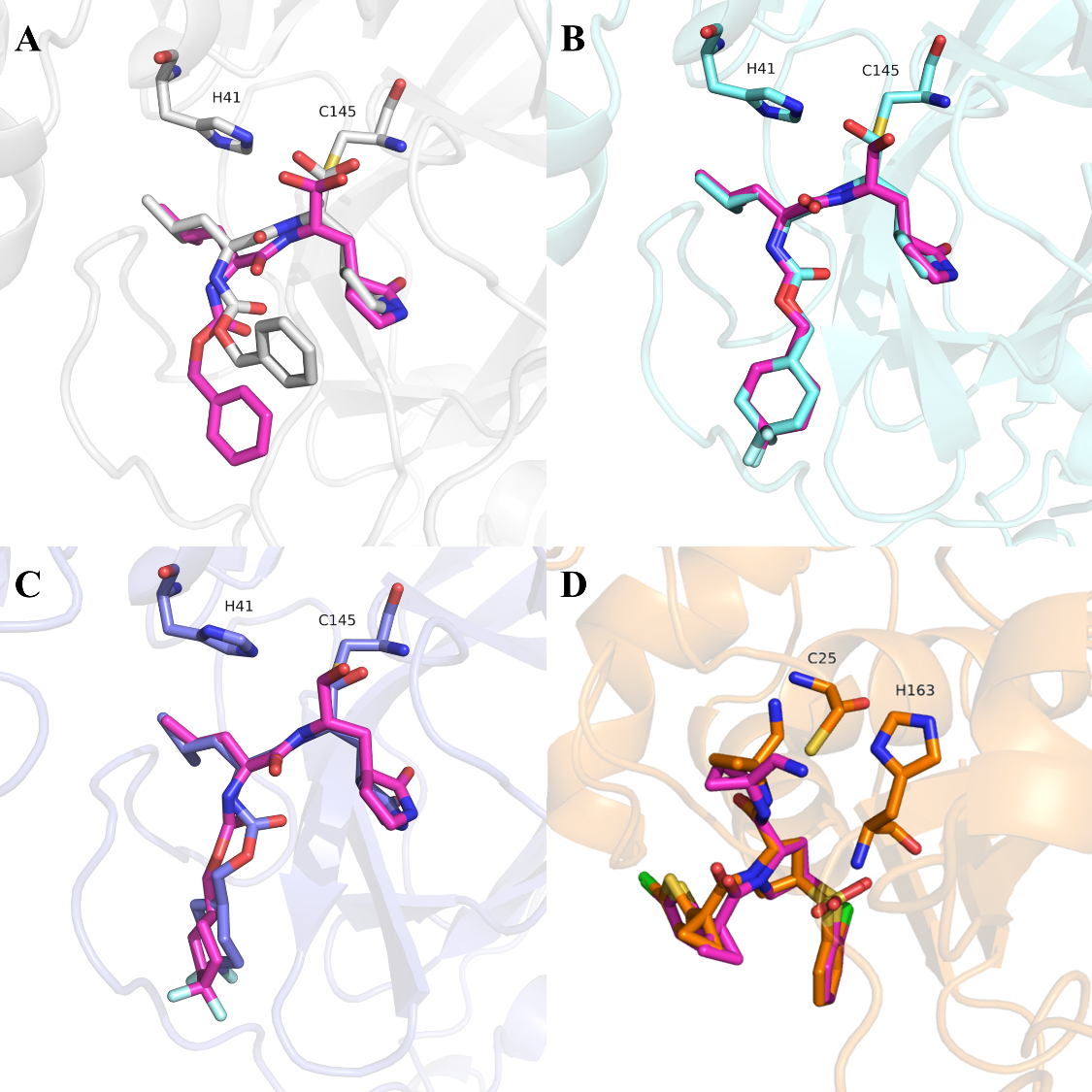


**Figure S5.** Redocking of M^pro^ and hCatL co-crystallized ligands using GOLD. Redocking poses (magenta) of the co-crystallized ligands, (A) GC376 (RMSD of 1.92 Å) in SARS-CoV-2 M^pro^ (white; PDB ID: 7D1M), (B) **7j** (RMSD of 0.73 Å) in SARS-CoV M^pro^ (aquamarine; PDB ID: 6W2A), (C) **7j** (RMSD of 1.49 Å) in MERS-CoV M^pro^ (slate blue; PDB ID: 6VH3), and (D) **XU3** (RMSD of 0.84 Å) in hCatL (orange; PDB ID: 2XU3). The catalytic residues His41 and Cys145 in M^pro^, and Cys25 and His163 in hCatL are displayed as sticks and labeled (black). Hydrogen atoms were hidden for clarity. Images were generated with PyMOL (v2.5.7).


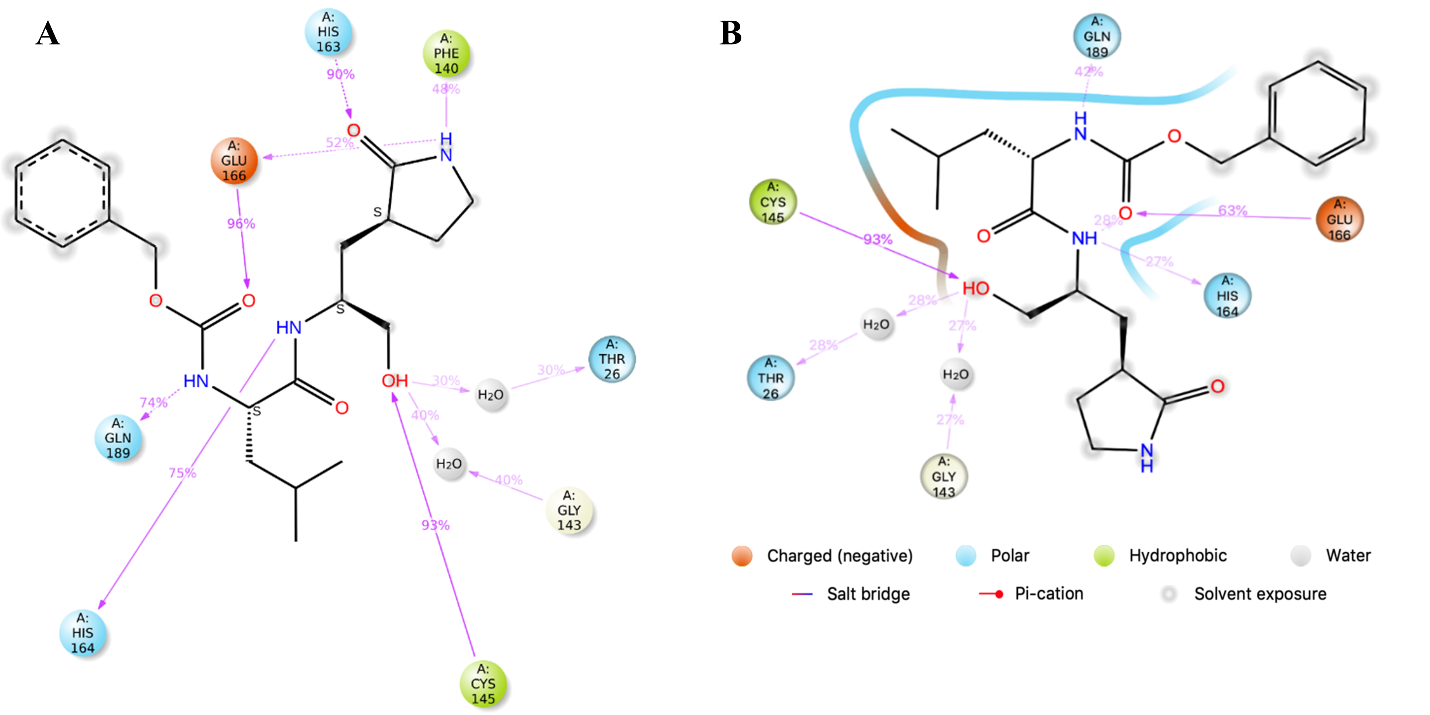


**Figure S6.** Noncovalent MD simulations of the co-crystallized ligand GC376 in SARS-CoV-2 M^pro^. Five replicates of 200 ns simulations were performed in two experimentally determined binding modes (A) PDB ID: 7D1M and (B) PDB ID: 7C6U. Similar interaction frequencies with amide groups are observed for Phe140, His163, His164, Glu166 and Gln189, in addition to interactions of Thr26, Gly143 and Cys145 with a P1 hydroxyl group. Interaction types and trend lines: hydrogen bonding or polar (blue), ionic or negatively charged (red), hydrophobic (green) and water-mediated (white). Images were generated using Schrödinger.


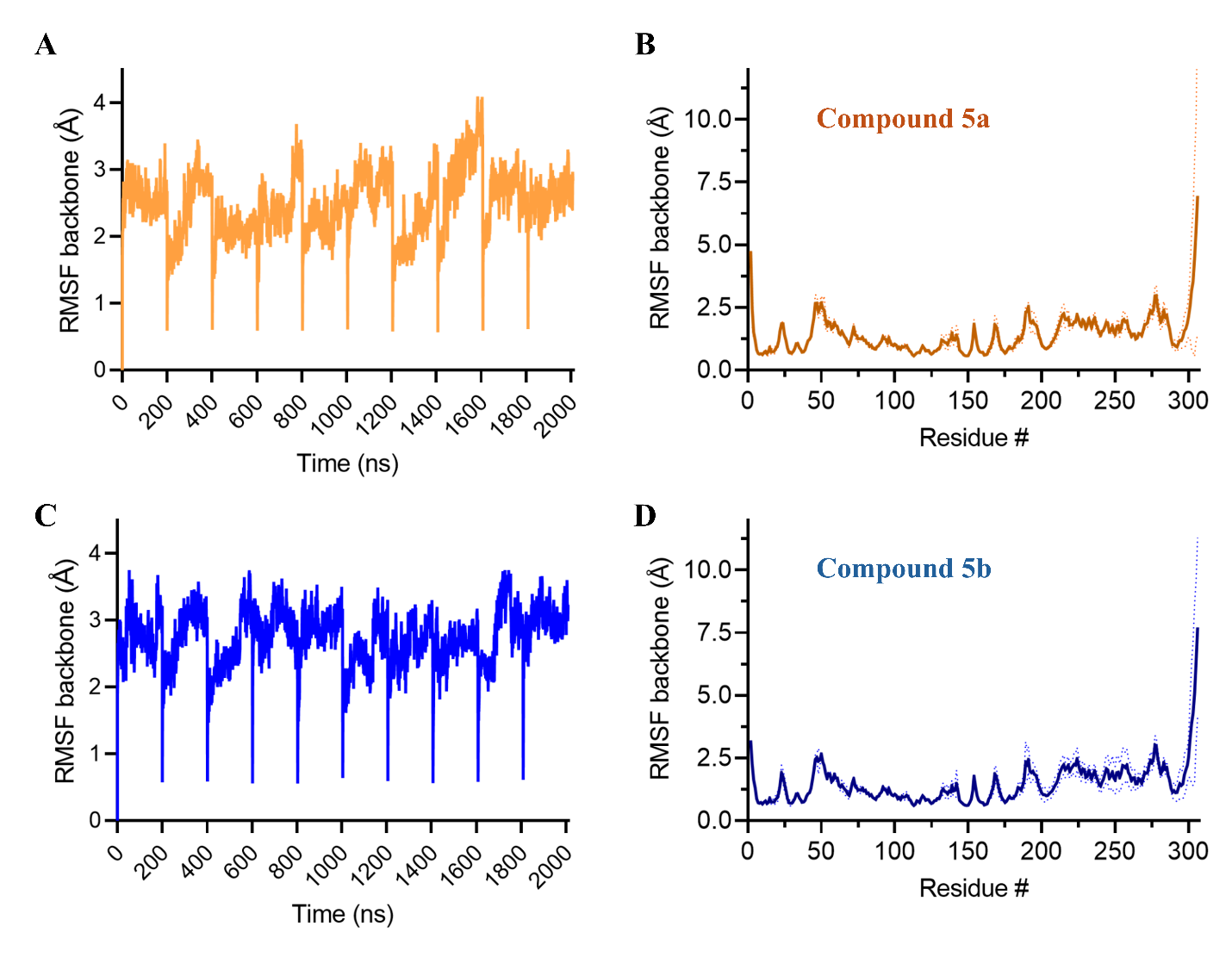


**Figure S7**. RMSD and RMSF values for **5a** (A and B, respectively) and **5b** (C and D, respectively). Variation of the values along the simulations (10 x 200 ns) are presented for both the template crystal structure’s backbone and the simulations with each compound. The changes in RMSF are normalized by residue for the protein backbone.


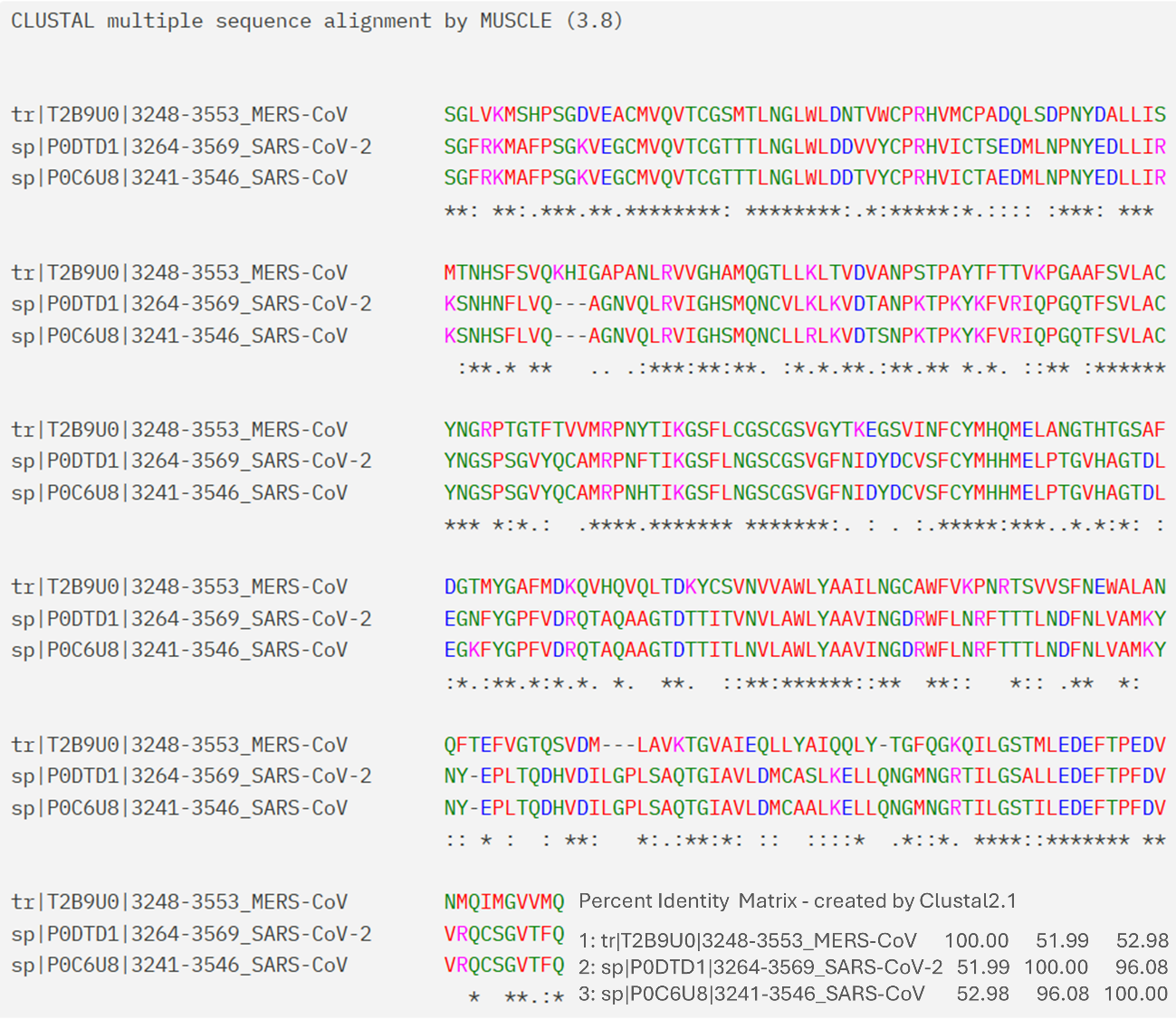


**Figure S8.** Alignment of different coronavirus M^pro^ sequences (SARS-CoV-2, SARS-CoV, and MERS-CoV). Sequences were retrieved from UniProt after being selected in PDB. Accession numbers: P0DTD1 (SARS-CoV-2; PDB ID 7D1M), P0C6U8 (SARS-CoV; PDB ID 6W2A) and T2B9U0 (MERS-CoV; PDB ID 6VH3). Sequences were aligned with MUSCLE from EMBL-EBI (available at <https://www.ebi.ac.uk/jdispatcher/msa/muscle>) resulting in the percent identity matrix (bottom right).

**Chemistry**

All solvents and reagents were purchased from reputable vendors and used as received. Thin layer chromatography was performed with 200 μM MilliporeSigma precoated silica gel aluminum sheets. Flash chromatography was performed with SiliaFlash P60 (particle size 40 – 63 μM) supplied by Silicycle. ^1^H and ^13^C NMR spectra were recorded on a 600 MHz NMR spectrometer. Chemical shifts were reported relative to residual solvent’s peak. All final compounds were found to be > 95% pure by LC/MS.

**Scheme 1.** *Reactions and conditions*. (a) *N-*Cbz-*L*-phenylalanine **i** (1.00 eq), NHS (1.00 eq), DCC (1.10 eq), CHCl_3_ (0.05 M), room temperature (rt), 1 h. (b) *D*-alanine (1.00 eq), NaHCO_3_ (2.00 eq.), H_2_O/acetone (1:2, 0.05 M), rt, 16 h (89% over two steps). (c) *N-*Cbz-*L*-phenylalanyl-*D*-alanine **ii** (1.00 eq), *N*,*O*-dimethyl-hydroxylamine hydrochloride (1.10 eq), ethyl chloroformate (1.00 eq), *N-*methylmorpholine (1.00 eq), freshly prepared diazomethane (6.00 eq), THF (0.1 M), -78 °C to rt, 16 h (37%). (d) Diazoketone **iii** (1.00 eq), freshly prepared DMDO (3.00 eq), acetone (0.02 M), -78 °C to rt, 16 h (99%). (e) Glyoxal **iv** (1.00 eq), methyl 2-(dimethoxyphosphoryl)acetate (1.10 eq), NaH (1.10 eq), CH_3_CN (0.05 M), 0 °C to rt, 16 h (12%).

***N-*Cbz-*L*-phenylalanyl-*D*-alanine** (**ii**) To a solution of *N-*Cbz-*L-*phenylalanine **i** (1.00 g, 3.34 mmol, 1.00 eq) and NHS (0.384 g, 3.34 mmol, 1.00 eq) in CHCl_3_ (60 mL), DCC (0.758 g, 3.67 mmol, 1.10 eq) was added at rt. The mixture was stirred at rt for 1 h. The precipitate was filtered off, and the filtrate was concentrated *en vacuo*. The resulting crude was used in the subsequent reaction without further purification. To a solution of the crude intermediate in acetone (80 mL), a solution of *D-*alanine (0.298 g, 3.34 mmol, 1.00 eq) and NaHCO_3_ (0.561 g, 6.68 mmol, 2.00 eq) in water (40 mL) was added at rt. The reaction mixture was stirred at rt. After 16 h, the mixture was concentrated *en vacuo* to remove the acetone. The resulting mixture was diluted with 60 mL water, and the remaining precipitate was removed by filtration. The filtrate was acidified to pH 3, and the resulting precipitate was collected by filtration (1.10 g, 3.00 mmol, 89%). ^1^H NMR (600 MHz, CDCl_3_) δ 7.37 – 7.27 (m, 4H), 7.27 – 7.09 (m, 7H), 6.74 (d, *J* = 7.1 Hz, 1H), 5.75 (d, *J* = 8.6 Hz, 1H), 5.06 (d, *J* = 12.2 Hz, 1H), 5.01 (d, *J* = 12.4 Hz, 1H), 4.61 (s, 1H), 4.46 (s, 1H), 3.03 (d, *J* = 7.5 Hz, 2H), 1.22 (d, *J* = 7.2 Hz, 3H) ppm. MS (ESI) [M+H]^+^  371.1.

**Benzyl ((*S*)-1-(((*R*)-4-diazo-3-oxobutan-2-yl)amino)-1-oxo-3-phenylpropan-2-yl)carbamate** (Diazoketone **iii**). To a solution of *N-*Cbz-*L*-phenylalanyl-*D*-alanine **ii** (0.500 g, 1.35 mmol, 1.00 eq) in anh. THF (13 mL), methylmorpholine (0.137 g, 1.35 mmol, 1.00 eq) was added, followed by ethyl chloroformate (0.146 g, 1.35 mmol, 1.00 eq). Freshly distilled solution of diazomethane (prepared using diazald and KOH in ethanol and water) was slowly added (4.0 mL) at -78 °C. The reaction mixture was slowly warmed to rt and stirred overnight. After 16 h, the reaction mixture was diluted with cold satd. NaHCO_3_ and extracted with EtOAc (3x). The organic extracts were combined and dried over Na_2_SO_4_. The crude mixture was concentrated *en vacuo* and purified via column chromatography (up to 50% EtOAc in hexanes) to yield the product as a white solid (0.490 g, 1.19 mmol, 88%). ^1^H NMR (600 MHz, DMSO-*d_6_*) δ 8.43 (d, J = 7.6 Hz, 1H), 7.60 (d, J = 8.1 Hz, 1H), 7.39 – 7.15 (m, 10H), 6.08 (s, 1H), 4.98 (d, J = 2.6 Hz, 2H), 4.32 – 4.17 (m, 2H), 2.93 (dd, J = 13.6, 5.2 Hz, 1H), 2.80 (dd, J = 13.4, 9.7 Hz, 1H), 2.51 (s, 2H), 1.13 (d, J = 7.3 Hz, 3H) ppm.

**Benzyl ((*S*)-1-(((*R*)-3,4-dioxobutan-2-yl)amino)-1-oxo-3-phenylpropan-2-yl)carbamate** (Glyoxal **iv**). To a solution of benzyl ((*S*)-1-(((*R*)-4-diazo-3-oxobutan-2-yl)amino)-1-oxo-3-phenylpropan-2-yl)carbamate **iii** (0.200 g, 0.507 mmol, 1.00 eq) in acetone (1 mL), freshly prepared DMDO (25 mL; prepared from 50 g Oxone and 48 g NaHCO_3_ in H_2_O/acetone) was added slowly at -78 °C. The reaction mixture was slowly warmed to rt and stirred overnight. After 16 h, the resulting mixture was concentrated *en vacuo* and used in the subsequent step without further purification (0.193 g, 0.505 mmol, 99%). MS (ESI) [M+H]^+^ 383.1

**Methyl (*R,E*)-5-((*S*)-2-(((benzyloxy)carbonyl)amino)-3-phenylpropanamido)-4-oxohex-2-enoate** (**1a**). The desired ylide was first prepared. To a solution of methyl 2-(dimethoxyphosphoryl)acetate (0.033 mg, 0.180 mmol, 1.00 eq) in anh. CH_3_CN (1.0 mL), NaH (0.0044 g, 0.180 mmol, 1.00 eq) was added. The resulting suspension was added to a solution of glyoxal **iv** (0.070 g, 0.180 mmol, 1.00 eq) in anh. CH_3_CN (1.0 mL) at 0 °C. The reaction mixture was slowly warmed to rt and stirred overnight. After 16 h, the solvent was concentrated *en vacuo* and the crude was purified via reverse-phase preparative HPLC as a white solid (0.010 g, 0.023 mmol, 12%). ^1^H NMR (600 MHz, CDCl_3_) δ 7.32 – 7.16 (m, 10H), 7.12 (d, *J* = 15.9 Hz, 1H), 6.80 (d, *J* = 15.7 Hz, 1H), 6.41 – 6.31 (m, 1H), 5.32 (s, 1H), 5.10 (s, 2H), 4.77 – 4.70 (m, 1H), 4.43 (s, 1H), 3.81 (s, 3H), 3.12 (s, 1H), 3.03 (dd, *J* = 13.6, 7.8 Hz, 1H), 1.20 (d, *J* = 7.3 Hz, 3H) ppm. MS (ESI) [M+H]^+^ 439.1.

**Scheme 2.** *Reactions and conditions*. (a) *N-*Cbz-*D*-phenylalanine **v** (1.00 eq), NHS (1.00 eq), DCC (1.10 eq), CHCl_3_ (0.05 M), rt, 1 h. (b) *D*-alanine (1.00 eq), NaHCO_3_ (2.00 eq.), H_2_O/acetone (1:2, 0.05 M), rt, 16 h (91% over two steps). (c) *N-*Cbz-*D*-phenylalanyl-*D*-alanine **vi** (1.00 eq), *N,O-*dimethyl-hydroxylamine hydrochloride (1.10 eq), HATU (1.10 eq), DIPEA (2.50 eq), DMF (0.1 M), rt, 16 h (88%). (d) Weinreb amide **vii** (1.00 eq), vinyl magnesium bromide (3.00 eq), THF (0.1 M), 0 °C to rt, 3 h (58%).

***N-*Cbz-*D*-phenylalanyl-*D*-alanine** (**vi**). The procedure for the synthesis of **ii** was closely followed using *N-*Cbz-*D-*phenylalanine **v** (1.00 g, 3.34 mmol, 1.00 eq), NHS (0.384 g, 3.34 mmol, 1.00 eq), DCC (0.758 g, 3.67 mmol, 1.10 eq), *D-*alanine (0.298 g, 3.34 mmol, 1.00 eq) and NaHCO_3_ (0.561 g, 6.68 mmol, 2.00 eq). MS (ESI) [M+H]^+^  371.1.

**Benzyl ((*R*)-1-(((*R*)-1-(methoxy(methyl)amino)-1-oxopropan-2-yl)amino)-1-oxo-3-phenylpropan-2-yl)carbamate** (Weinreb amine **vii**). To a solution of **vi** (0.500 g, 1.35 mmol, 1.00 eq) in anh. DMF (13 mL), *N,O-*dimethyl-hydroxylamine hydrochloride (0.145 g, 1.48 mmol, 1.10 eq) was added at rt, followed by HBTU (0.563 g, 1.48 mmol, 1.10 eq) and DIPEA (0.436 g, 3.37 mmol, 2.50 eq). The reaction mixture was stirred at rt for 16 h. The resulting mixture was diluted with water (20 mL) and extracted with EtOAc (3x). The organic extracts were combined and washed with water (5x), washed with brine, and dried over Na_2_SO_4_. The crude mixture was concentrated *en vacuo* and purified via column chromatography (up to 40% EtOAc/Hexanes) as a white solid (0.490 g, 1.19 mmol, 88%). ^1^H NMR (600 MHz, CDCl_3_) δ 7.82 – 7.66 (m, 10H), 5.55 – 5.50 (m, 2H), 5.36 – 5.30 (m, 1H), 4.92 (dd, *J* = 13.1, 7.1 Hz, 1H), 4.58 (q, *J* = 7.1 Hz, 2H), 4.20 (s, 3H), 3.63 (s, 3H), 3.53 (p, *J* = 5.8 Hz, 2H), 1.61 (d, *J* = 7.0 Hz, 3H) ppm. MS (ESI) [M+H]^+^  413.2.

**Benzyl ((*R*)-1-oxo-1-(((*R*)-3-oxopent-4-en-2-yl)amino)-3-phenylpropan-2-yl)carbamate** (**5a**) and **benzyl ((*R*)-1-oxo-1-(((*S*)-3-oxopent-4-en-2-yl)amino)-3-phenylpropan-2-yl)carbamate** (**5b**). To a solution of Weinreb amide **vii** (0.080 g, 0.190 mmol, 1.00 eq) in THF (2 mL), vinyl magnesium bromide (0.7 M in hexanes, 0.83 mL, 3.00 eq) was added at 0 °C. The reaction was slowly warmed to rt and stirred for 3 h. The resulting mixture was quenched with ice (20 mL) and extracted with EtOAc (3x). The combined organic extracts were combined and dried over Na_2_SO_4_. The crude mixture was concentrated *en vacuo* and purified via column chromatography (up to 30% EtOAc/Hexanes) as a mixture of two diastereomers (0.043 g, 0.110 mmol, 58%). The diastereomers were separated via chiral preparative HPLC. The experimental reports for crystal data and structure refinement for compounds **5a** and **5b** are available below in this supplemental file.

**Benzyl ((*R*)-1-oxo-1-(((*R*)-3-oxopent-4-en-2-yl)amino)-3-phenylpropan-2-yl)carbamate** (**5a**) ^1^H NMR (600 MHz, DMSO-d6) δ 8.46 (d, J = 7.1 Hz, 1H), 7.53 (d, J = 8.7 Hz, 1H), 7.35 – 7.16 (m, 9H), 6.63 (s, 0H), 6.48 (dd, J = 17.4, 10.6 Hz, 1H), 6.26 (dd, J = 17.4, 1.6 Hz, 1H), 5.83 (dd, J = 10.5, 1.5 Hz, 1H), 4.94 (s, 2H), 4.56 (t, J = 7.1 Hz, 1H), 4.28 (ddd, J = 10.8, 8.6, 4.3 Hz, 1H), 2.99 (dd, J = 13.8, 4.3 Hz, 1H), 2.73 (dd, J = 13.8, 10.6 Hz, 1H), 1.22 (d, J = 7.2 Hz, 3H) ppm. MS (ESI) [M+H]^+^  381.2. **Benzyl ((*R*)-1-oxo-1-(((*S*)-3-oxopent-4-en-2-yl)amino)-3-phenylpropan-2-yl)carbamate** (**5b**). ^1^H NMR (600 MHz, DMSO-*d*_6_) δ 8.42 (d, *J* = 7.3 Hz, 1H), 7.53 (d, *J* = 8.6 Hz, 1H), 7.37 – 7.18 (m, 9H), 6.70 (s, 0H), 6.57 (dd, *J* = 17.5, 10.6 Hz, 1H), 6.27 (dd, *J* = 17.5, 1.6 Hz, 1H), 5.83 (dd, *J* = 10.5, 1.6 Hz, 1H), 4.95 (s, 2H), 4.53 (t, *J* = 7.2 Hz, 1H), 4.33 – 4.25 (m, 1H), 2.94 (dd, *J* = 13.7, 4.9 Hz, 1H), 2.77 (dd, *J* = 13.7, 10.2 Hz, 1H), 1.13 (d, *J* = 7.2 Hz, 3H) ppm. MS (ESI) [M+H]^+^  381.2.

**Single Crystal Structure Reports**

Experimental Summary for Compound **5a** (CCDC 2248735)

The single crystal X-ray diffraction studies were carried out on a Bruker Microstar APEX II CCD diffractometer equipped with Cu K_α_ radiation (λ = 1.54178 Å).

Crystals of the subject compound were grown from MeOH/MTBE. A 0.23 x 0.07 x 0.03 mm piece of a crystal was mounted on a Cryoloop with Paratone oil. Data were collected in a nitrogen gas stream at 100(2) K using ϕ and ω scans. Crystal-to-detector distance was 40 mm and exposure time was 5, 20 or 45 seconds depending on the 2θ range per frame using a scan width of 1.25°. Data collection was 99.8 % complete to 67.679° in θ. A total of 24552 reflections were collected covering the indices, −5<=h<=5, −15<=k<=16, −34<=l<=32. 3666 reflections were found to be symmetry independent, with a R_int_ of 0.0469. Indexing and unit cell refinement indicated a **P**rimitive, **Orthorhombic** lattice. The space group was found to be ***P*2_1_2_1_2_1_***.* The data were integrated using the Bruker SAINT Software program and scaled using the SADABS software program. Solution by direct methods (SHELXT) produced a complete phasing model consistent with the proposed structure.

All nonhydrogen atoms were refined anisotropically by full-matrix least-squares (SHELXL-2014). All carbon bonded hydrogen atoms were placed using a riding model. Their positions were constrained relative to their parent atom using the appropriate HFIX command in SHELXL-2014. Crystallographic data are summarized in Table A.


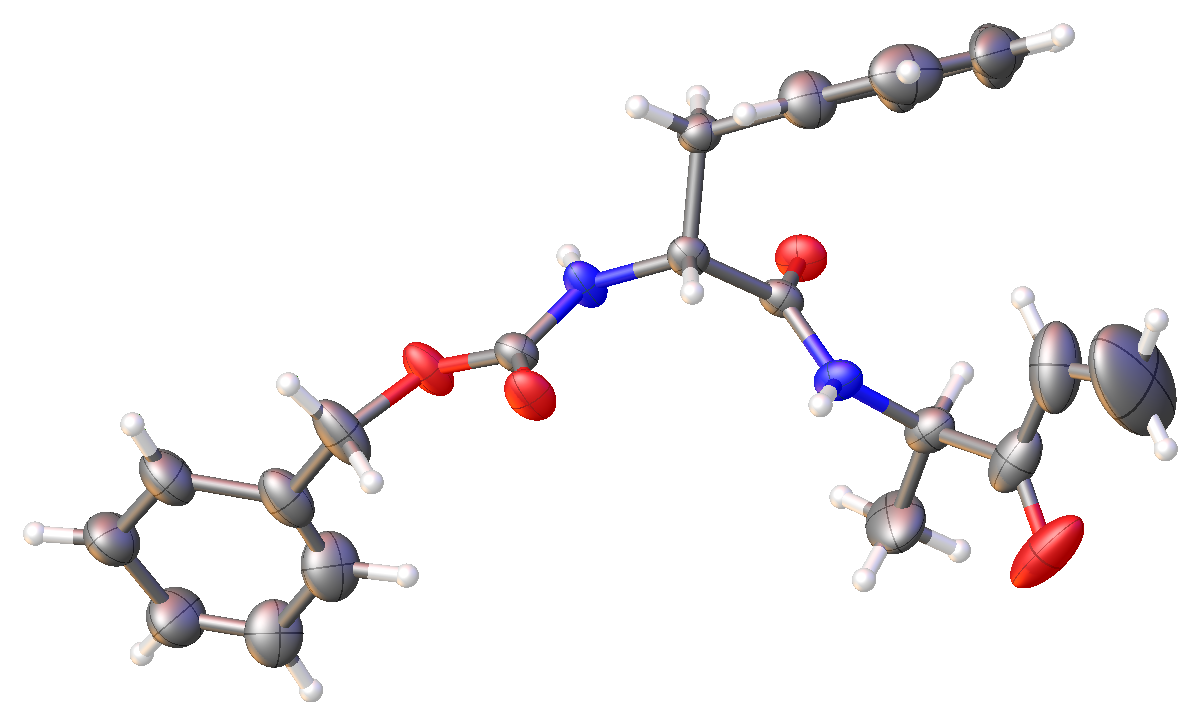
Table A. Crystal data and structure refinement for Compound **5a**.

Identification code 007f2_0m_a

Empirical formula C22 H24 N2 O4

Formula weight 380.43

Temperature 100.15 K

Wavelength 1.54178 Å

Crystal system Orthorhombic

Space group P2**_1_**2**_1_**2**_1_**

Unit cell dimensions a = 4.9628(2) Å α= 90°.

b = 13.9985(8) Å β= 90°.

c = 28.9752(14) Å γ = 90°.

Volume 2012.96(17) Å3

Z 4

Density (calculated) 1.255 Mg/m3

Absorption coefficient 0.707 mm-1

F(000) 808

Crystal size 0.23 x 0.07 x 0.03 mm3

Theta range for data collection 3.050 to 68.830°.

Index ranges -5<=h<=5, -15<=k<=16, -34<=l<=32

Reflections collected 24552

Independent reflections 3666 [R(int) = 0.0469]

Completeness to theta = 67.679° 99.8 %

Absorption correction Semi-empirical from equivalents

Max. and min. transmission 0.5851 and 0.4814

Refinement method Full-matrix least-squares on F2

Data / restraints / parameters 3666 / 72 / 289

Goodness-of-fit on F2 1.045

Final R indices [I>2sigma(I)] R1 = 0.0624, wR2 = 0.1685

R indices (all data) R1 = 0.0706, wR2 = 0.1774

Absolute structure parameter -0.08(12)

Extinction coefficient 0.0034(9)

Largest diff. peak and hole 0.633 and -0.323 e.Å-3

Table B. Atomic coordinates (x 104) and equivalent isotropic displacement parameters (Å2 x 103) for Compound **5a**. U(eq) is defined as one third of the trace of the orthogonalized Uij tensor.

________________________________________________________________________________

x y z U(eq)

________________________________________________________________________________

O(1) 9601(8) 5269(4) 2545(1) 79(1)

O(2) 2115(5) 5887(2) 3738(1) 34(1)

O(3) 9180(5) 5592(2) 4861(1) 38(1)

O(4) 5857(6) 4779(2) 5222(1) 40(1)

N(1) 6518(6) 5803(2) 3544(1) 31(1)

N(2) 4814(6) 5782(2) 4662(1) 29(1)

C(1) 9590(30) 7286(9) 2424(4) 165(6)

C(2) 8094(16) 6853(5) 2715(2) 79(2)

C(3) 8115(10) 5790(4) 2757(2) 51(1)

C(4) 6044(9) 5373(3) 3090(1) 37(1)

C(5) 6003(16) 4306(4) 3107(2) 68(2)

C(6) 4508(7) 6021(3) 3832(1) 27(1)

C(7) 5354(7) 6462(3) 4292(1) 26(1)

C(8) 3871(8) 7410(3) 4368(1) 30(1)

C(9) 4515(8) 8132(3) 4002(1) 32(1)

C(10) 6682(11) 8738(3) 4047(2) 51(1)

C(11) 7289(13) 9396(4) 3696(2) 68(2)

C(12) 5760(14) 9450(4) 3309(2) 60(2)

C(13) 3623(14) 8859(4) 3265(2) 62(2)

C(14) 3008(12) 8196(4) 3606(2) 49(1)

C(15) 6822(8) 5407(3) 4908(1) 27(1)

C(16) 7822(10) 4396(4) 5547(2) 57(1)

C(17) 7054(8) 3383(3) 5665(2) 49(1)

C(18A) 4434(8) 3155(4) 5794(3) 44(2)

C(19A) 3758(13) 2215(5) 5896(4) 62(3)

C(20A) 5700(20) 1502(4) 5869(5) 120(11)

C(21A) 8323(19) 1730(4) 5741(5) 113(7)

C(22A) 8999(11) 2670(5) 5639(4) 109(7)

C(18) 6680(20) 3224(7) 6171(4) 54(2)

C(19) 6160(20) 2305(7) 6302(3) 55(2)

C(20) 5850(30) 1551(8) 5925(5) 63(4)

C(21) 6390(20) 1752(8) 5519(4) 54(2)

C(22) 6890(20) 2720(8) 5389(4) 60(2)

________________________________________________________________________________ Table C. Bond lengths [Å] and angles [°] for Compound **5a**.

_____________________________________________________

O(1)-C(3) 1.205(7)

O(2)-C(6) 1.233(5)

O(3)-C(15) 1.207(5)

O(4)-C(15) 1.352(5)

O(4)-C(16) 1.458(5)

N(1)-H(1) 0.8800

N(1)-C(4) 1.465(5)

N(1)-C(6) 1.336(5)

N(2)-H(2) 0.8800

N(2)-C(7) 1.458(4)

N(2)-C(15) 1.334(5)

C(1)-H(1A) 0.9500

C(1)-H(1B) 0.9500

C(1)-C(2) 1.279(11)

C(2)-H(2A) 0.9500

C(2)-C(3) 1.494(9)

C(3)-C(4) 1.527(6)

C(4)-H(4) 1.0000

C(4)-C(5) 1.494(7)

C(5)-H(5A) 0.9800

C(5)-H(5B) 0.9800

C(5)-H(5C) 0.9800

C(6)-C(7) 1.526(5)

C(7)-H(7) 1.0000

C(7)-C(8) 1.535(5)

C(8)-H(8A) 0.9900

C(8)-H(8B) 0.9900

C(8)-C(9) 1.500(5)

C(9)-C(10) 1.377(7)

C(9)-C(14) 1.373(6)

C(10)-H(10) 0.9500

C(10)-C(11) 1.406(7)

C(11)-H(11) 0.9500

C(11)-C(12) 1.355(9)

C(12)-H(12) 0.9500

C(12)-C(13) 1.351(9)

C(13)-H(13) 0.9500

C(13)-C(14) 1.390(7)

C(14)-H(14) 0.9500

C(16)-H(16A) 0.9900

C(16)-H(16B) 0.9900

C(16)-C(17) 1.508(6)

C(17)-C(18A) 1.3900

C(17)-C(22A) 1.3900

C(17)-C(18) 1.492(11)

C(17)-C(22) 1.228(12)

C(18A)-H(18A) 0.9500

C(18A)-C(19A) 1.3900

C(19A)-H(19A) 0.9500

C(19A)-C(20A) 1.3900

C(20A)-H(20A) 0.9500

C(20A)-C(21A) 1.3900

C(21A)-H(21A) 0.9500

C(21A)-C(22A) 1.3900

C(22A)-H(22A) 0.9500

C(18)-H(18) 0.9500

C(18)-C(19) 1.367(13)

C(19)-H(19) 0.9500

C(19)-C(20) 1.529(17)

C(20)-H(20) 0.9500

C(20)-C(21) 1.240(16)

C(21)-H(21) 0.9500

C(21)-C(22) 1.429(14)

C(22)-H(22) 0.9500

C(15)-O(4)-C(16) 115.9(3)

C(4)-N(1)-H(1) 118.8

C(6)-N(1)-H(1) 118.8

C(6)-N(1)-C(4) 122.3(3)

C(7)-N(2)-H(2) 119.6

C(15)-N(2)-H(2) 119.6

C(15)-N(2)-C(7) 120.9(3)

H(1A)-C(1)-H(1B) 120.0

C(2)-C(1)-H(1A) 120.0

C(2)-C(1)-H(1B) 120.0

C(1)-C(2)-H(2A) 119.3

C(1)-C(2)-C(3) 121.5(10)

C(3)-C(2)-H(2A) 119.3

O(1)-C(3)-C(2) 124.4(6)

O(1)-C(3)-C(4) 120.2(5)

C(2)-C(3)-C(4) 115.3(5)

N(1)-C(4)-C(3) 107.6(3)

N(1)-C(4)-H(4) 107.3

N(1)-C(4)-C(5) 112.6(4)

C(3)-C(4)-H(4) 107.3

C(5)-C(4)-C(3) 114.3(4)

C(5)-C(4)-H(4) 107.3

C(4)-C(5)-H(5A) 109.5

C(4)-C(5)-H(5B) 109.5

C(4)-C(5)-H(5C) 109.5

H(5A)-C(5)-H(5B) 109.5

H(5A)-C(5)-H(5C) 109.5

H(5B)-C(5)-H(5C) 109.5

O(2)-C(6)-N(1) 123.1(3)

O(2)-C(6)-C(7) 121.3(3)

N(1)-C(6)-C(7) 115.6(3)

N(2)-C(7)-C(6) 109.1(3)

N(2)-C(7)-H(7) 108.6

N(2)-C(7)-C(8) 111.7(3)

C(6)-C(7)-H(7) 108.6

C(6)-C(7)-C(8) 110.1(3)

C(8)-C(7)-H(7) 108.6

C(7)-C(8)-H(8A) 109.2

C(7)-C(8)-H(8B) 109.2

H(8A)-C(8)-H(8B) 107.9

C(9)-C(8)-C(7) 112.2(3)

C(9)-C(8)-H(8A) 109.2

C(9)-C(8)-H(8B) 109.2

C(10)-C(9)-C(8) 120.9(4)

C(14)-C(9)-C(8) 121.3(4)

C(14)-C(9)-C(10) 117.7(4)

C(9)-C(10)-H(10) 119.9

C(9)-C(10)-C(11) 120.1(5)

C(11)-C(10)-H(10) 119.9

C(10)-C(11)-H(11) 119.5

C(12)-C(11)-C(10) 121.0(5)

C(12)-C(11)-H(11) 119.5

C(11)-C(12)-H(12) 120.5

C(13)-C(12)-C(11) 119.0(5)

C(13)-C(12)-H(12) 120.5

C(12)-C(13)-H(13) 119.6

C(12)-C(13)-C(14) 120.9(5)

C(14)-C(13)-H(13) 119.6

C(9)-C(14)-C(13) 121.2(5)

C(9)-C(14)-H(14) 119.4

C(13)-C(14)-H(14) 119.4

O(3)-C(15)-O(4) 124.0(3)

O(3)-C(15)-N(2) 125.4(3)

N(2)-C(15)-O(4) 110.6(3)

O(4)-C(16)-H(16A) 109.9

O(4)-C(16)-H(16B) 109.9

O(4)-C(16)-C(17) 108.8(4)

H(16A)-C(16)-H(16B) 108.3

C(17)-C(16)-H(16A) 109.9

C(17)-C(16)-H(16B) 109.9

C(18A)-C(17)-C(16) 120.9(4)

C(18A)-C(17)-C(22A) 120.0

C(22A)-C(17)-C(16) 119.1(4)

C(18)-C(17)-C(16) 113.1(6)

C(22)-C(17)-C(16) 125.4(6)

C(22)-C(17)-C(18) 121.3(7)

C(17)-C(18A)-H(18A) 120.0

C(17)-C(18A)-C(19A) 120.0

C(19A)-C(18A)-H(18A) 120.0

C(18A)-C(19A)-H(19A) 120.0

C(20A)-C(19A)-C(18A) 120.0

C(20A)-C(19A)-H(19A) 120.0

C(19A)-C(20A)-H(20A) 120.0

C(19A)-C(20A)-C(21A) 120.0

C(21A)-C(20A)-H(20A) 120.0

C(20A)-C(21A)-H(21A) 120.0

C(22A)-C(21A)-C(20A) 120.0

C(22A)-C(21A)-H(21A) 120.0

C(17)-C(22A)-H(22A) 120.0

C(21A)-C(22A)-C(17) 120.0

C(21A)-C(22A)-H(22A) 120.0

C(17)-C(18)-H(18) 122.0

C(19)-C(18)-C(17) 115.9(9)

C(19)-C(18)-H(18) 122.0

C(18)-C(19)-H(19) 121.0

C(18)-C(19)-C(20) 118.0(9)

C(20)-C(19)-H(19) 121.0

C(19)-C(20)-H(20) 120.0

C(21)-C(20)-C(19) 120.0(10)

C(21)-C(20)-H(20) 120.0

C(20)-C(21)-H(21) 119.9

C(20)-C(21)-C(22) 120.2(11)

C(22)-C(21)-H(21) 119.9

C(17)-C(22)-C(21) 123.8(9)

C(17)-C(22)-H(22) 118.1

C(21)-C(22)-H(22) 118.1

Table D. Anisotropic displacement parameters (Å2 x 103) for Compound **5a**. The anisotropic displacement factor exponent takes the form: -2π2 [h2 a*2U11 + ... + 2 h k a* b* U12]

______________________________________________________________________________

U11 U22 U33 U23 U13 U12

______________________________________________________________________________

O(1) 43(2) 135(4) 60(2) -48(2) 12(2) -7(2)

O(2) 18(1) 41(2) 43(2) -2(1) -4(1) -3(1)

O(3) 17(1) 47(2) 50(2) 13(1) -2(1) -1(1)

O(4) 24(1) 40(2) 54(2) 22(1) -6(1) -3(1)

N(1) 19(2) 37(2) 36(2) -7(1) -3(1) 1(1)

N(2) 15(2) 35(2) 38(2) 13(1) -1(1) -3(1)

C(1) 140(10) 176(11) 179(10) 115(9) 42(8) -13(8)

C(2) 91(5) 95(5) 51(3) 17(3) -5(3) -44(4)

C(3) 35(2) 80(4) 37(2) -15(2) 1(2) -15(2)

C(4) 29(2) 43(2) 38(2) -8(2) -2(2) 0(2)

C(5) 108(5) 45(3) 52(3) -16(2) -6(3) -3(3)

C(6) 19(2) 25(2) 36(2) 4(1) -2(1) 0(1)

C(7) 17(2) 29(2) 32(2) 4(1) 2(1) -4(1)

C(8) 29(2) 28(2) 32(2) 0(1) 4(2) 1(2)

C(9) 32(2) 27(2) 37(2) 1(2) 10(2) 4(2)

C(10) 53(3) 41(3) 60(3) 5(2) 5(2) -10(2)

C(11) 64(4) 41(3) 98(5) 10(3) 27(3) -13(2)

C(12) 80(4) 41(3) 57(3) 17(2) 29(3) 9(3)

C(13) 88(4) 54(3) 45(3) 19(2) 7(3) 1(3)

C(14) 59(3) 47(3) 43(2) 13(2) -3(2) -4(2)

C(15) 23(2) 24(2) 34(2) 3(1) 0(2) -1(1)

C(16) 37(3) 58(3) 76(3) 35(3) -15(2) -6(2)

C(17) 34(2) 49(2) 63(2) 21(2) -7(2) -5(2)

C(18A) 32(3) 36(4) 65(6) 25(3) -9(3) -2(2)

C(19A) 56(5) 39(4) 89(8) 31(4) -14(5) -11(3)

C(20A) 94(7) 60(5) 210(30) 71(8) 38(9) 19(4)

C(21A) 93(7) 69(5) 178(19) 62(7) 34(9) 16(4)

C(22A) 66(5) 70(4) 192(19) 65(6) 47(7) 24(4)

C(18) 59(5) 42(3) 62(3) 17(2) -4(3) 2(3)

C(19) 67(6) 40(3) 59(4) 12(2) 8(3) 4(3)

C(20) 85(9) 41(4) 65(4) 9(3) 14(4) -4(4)

C(21) 47(6) 57(3) 59(4) 6(2) 2(3) -22(3)

C(22) 58(6) 59(3) 64(3) 13(2) 5(3) -22(3)

______________________________________________________________________________ Table E. Hydrogen coordinates (x 104) and isotropic displacement parameters (Å2 x 10 3) for Compound **5a**.

________________________________________________________________________________

x y z U(eq)

________________________________________________________________________________

H(1) 8186 5921 3630 37

H(2) 3140 5617 4724 35

H(1A) 10756 6930 2228 198

H(1B) 9548 7963 2403 198

H(2A) 6941 7216 2910 95

H(4) 4225 5587 2984 44

H(5A) 7767 4071 3208 102

H(5B) 5602 4054 2799 102

H(5C) 4615 4093 3324 102

H(7) 7335 6591 4282 31

H(8A) 1905 7292 4371 36

H(8B) 4380 7673 4674 36

H(10) 7770 8712 4317 61

H(11) 8796 9808 3730 81

H(12) 6184 9897 3073 72

H(13) 2527 8898 2996 75

H(14) 1517 7779 3564 59

H(16A) 7851 4791 5831 68

H(16B) 9644 4409 5408 68

H(18A) 3105 3642 5812 53

H(19A) 1967 2059 5984 74

H(20A) 5241 860 5939 144

H(21A) 9653 1243 5723 136

H(22A) 10790 2826 5551 131

H(18) 6806 3732 6387 65

H(19) 5998 2140 6619 67

H(20) 5233 928 6002 76

H(21) 6473 1260 5293 65

H(22) 7105 2860 5070 72

________________________________________________________________________________

Experimental Summary for Compound **5b** (CCDC 2244033)

The single crystal X-ray diffraction studies were carried out on a Bruker SMART Pt135 CCD diffractometer equipped with Cu K_α_ radiation (λ = 1.54178 Å).

Crystals of the subject compound were grown from DCM/Pentane. A 0.23 x 0.03 x 0.01 mm piece of a crystal was mounted on a Cryoloop with Paratone oil. Data were collected in a nitrogen gas stream at 100(2) K using ϕ and ω scans. Crystal-to-detector distance was 45 mm and exposure time was 5, 20, 30, or 45 seconds depending on the 2θ range per frame using a scan width of 1.25°. Data collection was 99.9 % complete to 66.682° in θ. A total of 15861 reflections were collected covering the indices, −16<=h<=15, −5<=k<=5, −17<=l<=15. 3462 reflections were found to be symmetry independent, with a R_int_ of 0.0519. Indexing and unit cell refinement indicated a **P**rimitive, **Monoclinic** lattice. The space group was found to be ***P*2_1_***.* The data were integrated using the Bruker SAINT Software program and scaled using the SADABS software program. Solution by direct methods (SHELXT) produced a complete phasing model consistent with the proposed structure.

All nonhydrogen atoms were refined anisotropically by full-matrix least-squares (SHELXL-2014). All carbon bonded hydrogen atoms were placed using a riding model. Their positions were constrained relative to their parent atom using the appropriate HFIX command in SHELXL-2014. Crystallographic data are summarized in Table A.


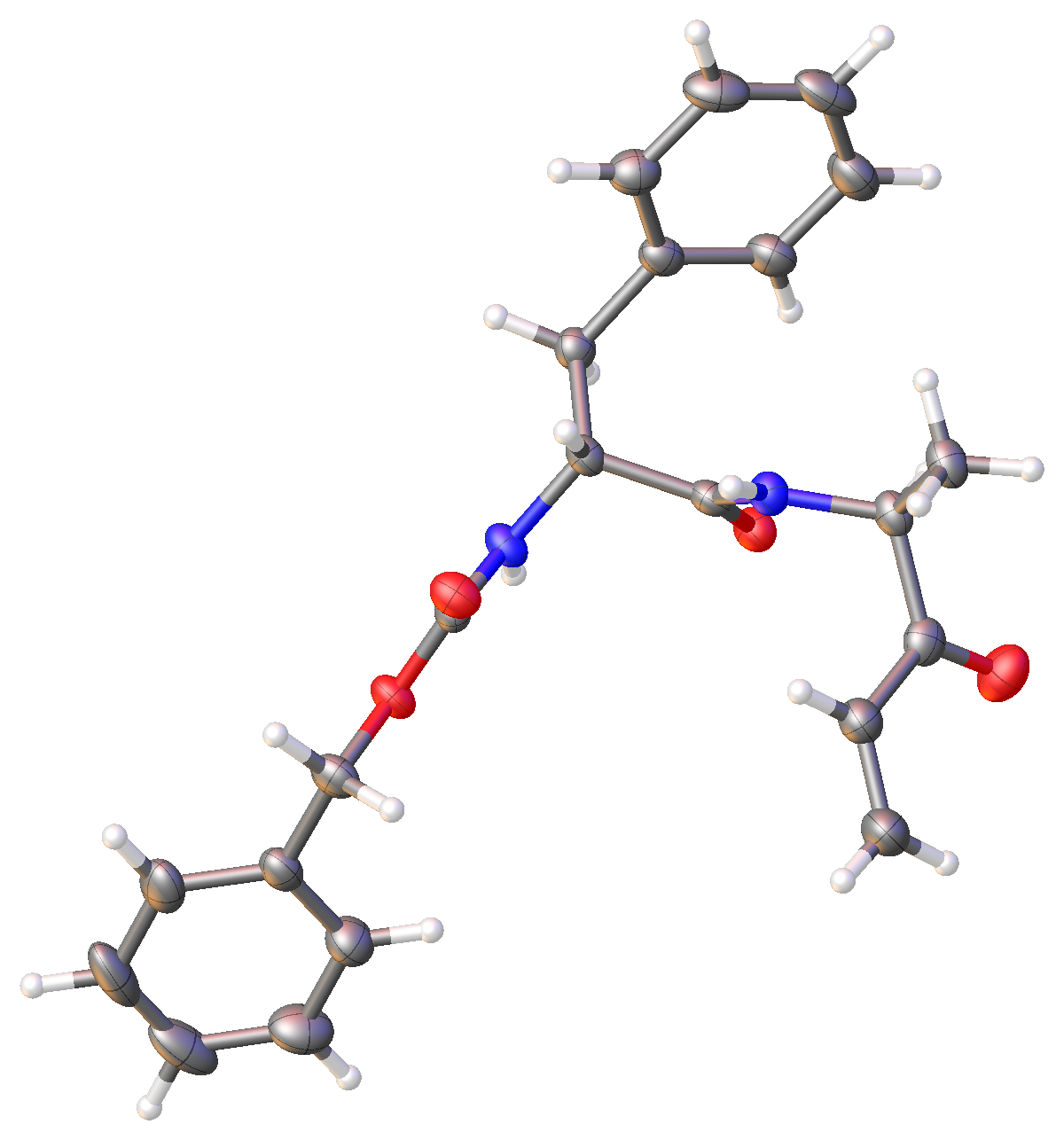


Table A. Crystal data and structure refinement for Compound **5b**.

Identification code 007f1_0m_a

Empirical formula C22 H24 N2 O4

Formula weight 380.43

Temperature 100.15 K

Wavelength 1.54178 Å

Crystal system Monoclinic

Space group P 1 21 1

Unit cell dimensions a = 13.625(3) Å α= 90°.

b = 4.9844(13) Å β= 94.307(9)°.

c = 14.574(4) Å γ = 90°.

Volume 987.0(4) Å3

Z 2

Density (calculated) 1.280 Mg/m3

Absorption coefficient 0.720 mm-1

F(000) 404

Crystal size 0.23 x 0.03 x 0.01 mm3

Theta range for data collection 3.041 to 66.682°.

Index ranges -16<=h<=15, -5<=k<=5, -17<=l<=15

Reflections collected 15861

Independent reflections 3462 [R(int) = 0.0519]

Completeness to theta = 66.682° 99.9 %

Absorption correction Semi-empirical from equivalents

Max. and min. transmission 0.5201 and 0.4238

Refinement method Full-matrix least-squares on F2

Data / restraints / parameters 3462 / 3 / 260

Goodness-of-fit on F2 1.052

Final R indices [I>2sigma(I)] R1 = 0.0381, wR2 = 0.0955

R indices (all data) R1 = 0.0431, wR2 = 0.0989

Absolute structure parameter -0.1(2)

Largest diff. peak and hole 0.125 and -0.186 e.Å-3

Table B. Atomic coordinates (x 104) and equivalent isotropic displacement parameters (Å2 x 103) for Compound **5b**. U(eq) is defined as one third of the trace of the orthogonalized Uij tensor.

________________________________________________________________________________

x y z U(eq)

________________________________________________________________________________

O(1) 4984(2) 3371(5) 2890(2) 41(1)

O(2) 2658(1) 1723(4) 3806(1) 27(1)

O(3) 2031(1) 8328(4) 5817(1) 30(1)

O(4) 2261(1) 4860(4) 6820(1) 27(1)

N(1) 2715(2) 6100(5) 3405(2) 25(1)

N(2) 1686(2) 4004(5) 5385(2) 25(1)

C(1) 5738(2) 6917(9) 4306(2) 44(1)

C(2) 4834(2) 7034(7) 3915(2) 34(1)

C(3) 4472(2) 5144(6) 3178(2) 29(1)

C(4) 3437(2) 5611(6) 2732(2) 26(1)

C(5) 3465(2) 7981(6) 2066(2) 32(1)

C(6) 2345(2) 4049(5) 3862(2) 23(1)

C(7) 1468(2) 4671(6) 4421(2) 24(1)

C(8) 569(2) 3063(6) 4013(2) 26(1)

C(9) 359(2) 3587(6) 2995(2) 27(1)

C(10) -255(2) 5698(6) 2689(2) 35(1)

C(11) -406(3) 6238(7) 1751(2) 43(1)

C(12) 57(3) 4740(8) 1119(2) 44(1)

C(13) 664(2) 2623(7) 1414(2) 41(1)

C(14) 802(2) 2047(7) 2345(2) 33(1)

C(15) 2004(2) 5927(6) 5991(2) 24(1)

C(16) 2651(2) 6776(6) 7516(2) 31(1)

C(17) 2963(2) 5200(6) 8365(2) 30(1)

C(18) 2528(2) 5652(7) 9183(2) 40(1)

C(19) 2843(3) 4231(9) 9969(2) 52(1)

C(20) 3588(3) 2341(8) 9944(2) 54(1)

C(21) 4013(3) 1854(8) 9129(2) 47(1)

C(22) 3704(2) 3283(7) 8345(2) 38(1)

________________________________________________________________________________ Table C. Bond lengths [Å] and angles [°] for Compound **5b**.

O(1)-C(3) 1.219(4)

O(2)-C(6) 1.240(3)

O(3)-C(15) 1.224(4)

O(4)-C(15) 1.342(3)

O(4)-C(16) 1.464(3)

N(1)-H(1) 0.881(13)

N(1)-C(4) 1.460(3)

N(1)-C(6) 1.339(4)

N(2)-H(2) 0.880(13)

N(2)-C(7) 1.452(3)

N(2)-C(15) 1.353(4)

C(1)-H(1A) 0.9500

C(1)-H(1B) 0.9500

C(1)-C(2) 1.319(4)

C(2)-H(2A) 0.9500

C(2)-C(3) 1.485(4)

C(3)-C(4) 1.525(4)

C(4)-H(4) 1.0000

C(4)-C(5) 1.531(4)

C(5)-H(5A) 0.9800

C(5)-H(5B) 0.9800

C(5)-H(5C) 0.9800

C(6)-C(7) 1.528(4)

C(7)-H(7) 1.0000

C(7)-C(8) 1.545(4)

C(8)-H(8A) 0.9900

C(8)-H(8B) 0.9900

C(8)-C(9) 1.513(4)

C(9)-C(10) 1.396(4)

C(9)-C(14) 1.392(4)

C(10)-H(10) 0.9500

C(10)-C(11) 1.393(5)

C(11)-H(11) 0.9500

C(11)-C(12) 1.375(5)

C(12)-H(12) 0.9500

C(12)-C(13) 1.388(5)

C(13)-H(13) 0.9500

C(13)-C(14) 1.385(4)

C(14)-H(14) 0.9500

C(16)-H(16A) 0.9900

C(16)-H(16B) 0.9900

C(16)-C(17) 1.500(4)

C(17)-C(18) 1.389(4)

C(17)-C(22) 1.392(4)

C(18)-H(18) 0.9500

C(18)-C(19) 1.386(5)

C(19)-H(19) 0.9500

C(19)-C(20) 1.388(6)

C(20)-H(20) 0.9500

C(20)-C(21) 1.382(6)

C(21)-H(21) 0.9500

C(21)-C(22) 1.385(5)

C(22)-H(22) 0.9500

C(15)-O(4)-C(16) 114.9(2)

C(4)-N(1)-H(1) 120(2)

C(6)-N(1)-H(1) 119(2)

C(6)-N(1)-C(4) 120.3(2)

C(7)-N(2)-H(2) 118(2)

C(15)-N(2)-H(2) 118(2)

C(15)-N(2)-C(7) 120.1(2)

H(1A)-C(1)-H(1B) 120.0

C(2)-C(1)-H(1A) 120.0

C(2)-C(1)-H(1B) 120.0

C(1)-C(2)-H(2A) 118.9

C(1)-C(2)-C(3) 122.2(3)

C(3)-C(2)-H(2A) 118.9

O(1)-C(3)-C(2) 122.7(3)

O(1)-C(3)-C(4) 119.9(3)

C(2)-C(3)-C(4) 117.2(3)

N(1)-C(4)-C(3) 112.8(2)

N(1)-C(4)-H(4) 108.3

N(1)-C(4)-C(5) 110.3(2)

C(3)-C(4)-H(4) 108.3

C(3)-C(4)-C(5) 108.7(2)

C(5)-C(4)-H(4) 108.3

C(4)-C(5)-H(5A) 109.5

C(4)-C(5)-H(5B) 109.5

C(4)-C(5)-H(5C) 109.5

H(5A)-C(5)-H(5B) 109.5

H(5A)-C(5)-H(5C) 109.5

H(5B)-C(5)-H(5C) 109.5

O(2)-C(6)-N(1) 122.5(3)

O(2)-C(6)-C(7) 120.7(2)

N(1)-C(6)-C(7) 116.7(2)

N(2)-C(7)-C(6) 111.0(2)

N(2)-C(7)-H(7) 108.9

N(2)-C(7)-C(8) 110.6(2)

C(6)-C(7)-H(7) 108.9

C(6)-C(7)-C(8) 108.6(2)

C(8)-C(7)-H(7) 108.9

C(7)-C(8)-H(8A) 109.2

C(7)-C(8)-H(8B) 109.2

H(8A)-C(8)-H(8B) 107.9

C(9)-C(8)-C(7) 111.9(2)

C(9)-C(8)-H(8A) 109.2

C(9)-C(8)-H(8B) 109.2

C(10)-C(9)-C(8) 120.6(3)

C(14)-C(9)-C(8) 120.9(3)

C(14)-C(9)-C(10) 118.5(3)

C(9)-C(10)-H(10) 120.0

C(11)-C(10)-C(9) 120.0(3)

C(11)-C(10)-H(10) 120.0

C(10)-C(11)-H(11) 119.7

C(12)-C(11)-C(10) 120.7(3)

C(12)-C(11)-H(11) 119.7

C(11)-C(12)-H(12) 120.1

C(11)-C(12)-C(13) 119.9(3)

C(13)-C(12)-H(12) 120.1

C(12)-C(13)-H(13) 120.2

C(14)-C(13)-C(12) 119.6(3)

C(14)-C(13)-H(13) 120.2

C(9)-C(14)-H(14) 119.4

C(13)-C(14)-C(9) 121.3(3)

C(13)-C(14)-H(14) 119.4

O(3)-C(15)-O(4) 124.3(3)

O(3)-C(15)-N(2) 124.8(3)

O(4)-C(15)-N(2) 110.9(2)

O(4)-C(16)-H(16A) 110.3

O(4)-C(16)-H(16B) 110.3

O(4)-C(16)-C(17) 107.2(2)

H(16A)-C(16)-H(16B) 108.5

C(17)-C(16)-H(16A) 110.3

C(17)-C(16)-H(16B) 110.3

C(18)-C(17)-C(16) 120.7(3)

C(18)-C(17)-C(22) 119.1(3)

C(22)-C(17)-C(16) 120.2(3)

C(17)-C(18)-H(18) 120.0

C(19)-C(18)-C(17) 120.1(3)

C(19)-C(18)-H(18) 120.0

C(18)-C(19)-H(19) 119.8

C(18)-C(19)-C(20) 120.5(3)

C(20)-C(19)-H(19) 119.8

C(19)-C(20)-H(20) 120.2

C(21)-C(20)-C(19) 119.7(3)

C(21)-C(20)-H(20) 120.2

C(20)-C(21)-H(21) 120.0

C(20)-C(21)-C(22) 119.9(4)

C(22)-C(21)-H(21) 120.0

C(17)-C(22)-H(22) 119.6

C(21)-C(22)-C(17) 120.7(3)

C(21)-C(22)-H(22) 119.6

Table D. Anisotropic displacement parameters (Å2 x 103) for Compound **5b**. The anisotropic displacement factor exponent takes the form: -2π2 [h2 a*2U11 + ... + 2 h k a* b* U12]

______________________________________________________________________________

U11 U22 U33 U23 U13 U12

______________________________________________________________________________

O(1) 36(1) 32(1) 55(1) -6(1) 10(1) 3(1)

O(2) 33(1) 22(1) 24(1) 0(1) 2(1) 2(1)

O(3) 40(1) 21(1) 29(1) 2(1) -2(1) -2(1)

O(4) 37(1) 24(1) 19(1) 0(1) -2(1) -2(1)

N(1) 29(1) 21(1) 24(1) -1(1) 4(1) 1(1)

N(2) 37(1) 20(1) 18(1) 2(1) 0(1) 1(1)

C(1) 40(2) 61(2) 29(2) -3(2) 0(1) 4(2)

C(2) 34(2) 40(2) 27(2) 0(1) 2(1) 2(1)

C(3) 32(1) 27(1) 29(1) 5(1) 8(1) -1(1)

C(4) 34(2) 22(1) 22(1) -1(1) 4(1) 1(1)

C(5) 43(2) 29(2) 24(1) 2(1) 6(1) 2(1)

C(6) 27(1) 23(1) 18(1) 0(1) -3(1) 1(1)

C(7) 30(1) 24(1) 19(1) 1(1) 2(1) -1(1)

C(8) 28(1) 27(1) 24(1) 2(1) 3(1) -2(1)

C(9) 27(1) 29(2) 25(1) 2(1) -2(1) -5(1)

C(10) 34(2) 33(2) 37(2) 5(1) -2(1) 0(1)

C(11) 45(2) 39(2) 43(2) 13(2) -14(1) -2(1)

C(12) 54(2) 50(2) 26(2) 11(2) -11(1) -15(2)

C(13) 48(2) 49(2) 26(2) -4(1) -1(1) -7(2)

C(14) 35(2) 36(2) 28(2) -2(1) -5(1) -2(1)

C(15) 25(1) 26(2) 21(1) 0(1) 4(1) 2(1)

C(16) 38(2) 28(2) 25(1) -4(1) -3(1) -3(1)

C(17) 32(1) 34(2) 22(1) -2(1) 0(1) -6(1)

C(18) 43(2) 46(2) 30(2) -13(2) 6(1) -9(2)

C(19) 72(2) 66(3) 20(2) -4(2) 8(2) -28(2)

C(20) 75(3) 55(2) 29(2) 15(2) -11(2) -26(2)

C(21) 50(2) 44(2) 45(2) 16(2) -7(2) -1(2)

C(22) 41(2) 42(2) 31(2) 5(2) 3(1) 2(1)

______________________________________________________________________________ Table E. Hydrogen coordinates (x 104) and isotropic displacement parameters (Å2 x 10 3) for Compound **5b**.

________________________________________________________________________________

x y z U(eq)

________________________________________________________________________________

H(1) 2580(20) 7760(40) 3560(20) 30

H(2) 1830(20) 2330(30) 5530(20) 30

H(1A) 6182 5592 4118 52

H(1B) 5945 8158 4776 52

H(2A) 4400 8372 4112 40

H(4) 3231 3975 2370 31

H(5A) 2799 8336 1790 48

H(5B) 3899 7549 1581 48

H(5C) 3714 9575 2402 48

H(7) 1316 6630 4369 29

H(8A) 693 1123 4112 32

H(8B) -16 3557 4340 32

H(10) -569 6767 3120 42

H(11) -834 7656 1546 52

H(12) -38 5153 483 53

H(13) 983 1577 980 49

H(14) 1205 572 2543 40

H(16A) 2138 8098 7650 37

H(16B) 3219 7752 7294 37

H(18) 2015 6936 9205 47

H(19) 2546 4554 10527 63

H(20) 3805 1385 10486 65

H(21) 4516 542 9107 56

H(22) 4001 2950 7787 45

________________________________________________________________________________
